# Supplementary material for: Microplastic pollution induces algae blooms in experimental ponds but bioplastics are less harmful
Source: Commun Sustain. 2026 Jan 19;1(1):16. doi: 10.1038/s44458-025-00014-6 (PMC12863645; doi:10.1038/s44458-025-00014-6)
Supplement: Supplementary file 2 — Supplementary information files [file 44458_2025_14_MOESM2_ESM.pdf]

Supplemental information:

Table of Contents

Contents	*Page #*
Supplementary Methods	
Synthesis method for TPU 181	2
Supplementary Figures	
Supplementary Figure 1	3
Supplementary Figure 2	4
Supplementary Figure 3	5
Supplementary Figure 4	6
Supplementary Figure 5	7
Supplementary Figure 6	8
Supplementary Figure 7	9
Supplementary Figure 8	10
Supplementary Figure 9	11
Supplementary Figure 10	12
Supplementary Figure 11	13
Supplementary Figure 12	14
Supplementary Figure 13	15
Supplementary Figure 14	16
Supplementary Figure 15	17
Supplementary Figure 16	18
Supplementary Tables	
Supplementary Table 1	19
Supplementary Table 2	20
Supplementary Table 3	21
Supplementary Table 4	22
Supplementary Table 5	23-24
Supplementary Table 6	25
Supplementary Table 7	26-27
Supplementary Table 8	28
Supplementary Table 9	29

Supplementary methods

Synthesis method for TPU 181

Algae-Based Polyester-Polyol Synthesis

The polyester-polyol used for TPU 181 was synthesized following Rajput *et al.* with modifications (Rajput et al. 2022). Briefly, a three-neck round-bottom flask equipped with a Dean–Stark apparatus, reflux condenser, and oil bath was purged and maintained under nitrogen. Commercial azelaic acid (356.4 g, Croda Inc.) and algae-sourced azelaic acid (40.3 g) were combined with 1,3-propanediol (179.2 g) as described in Phung et al. 2020. The mixture was heated to 150–160 °C and gradually raised to 180 °C over one hour. Within the first 4–5 h, water by-product was continuously removed. When approximately 80% of the water was collected, dibutyltin dilaurate (DBTDL, 0.04 wt %) was added. The reaction proceeded for an additional 2–3 days (until desired acid and hydroxyl numbers were reached), with periodic sampling for titration.

TPU 181 Preparation

The algae-based polyester-polyol (230 g) and 1,3-propanediol (8.74 g) were dried under vacuum at room temperature for 24 h before polymerization. These were then combined with DBTDL (0.03 wt %) and preheated (75 °C) hexamethylene diisocyanate (6HDI, 42.55 g) in a speed mixer (FlackTek) at 2000 rpm for approximately 1 min. The reaction mixture was poured into molds and cured at 75 °C for 2 days to form TPU sheets.

Mechanical Property Testing

Shore A hardness testing was conducted at room temperature. The resulting TPU 181 displayed a Shore A hardness of approximately 97, consistent with typical thermoplastic polyurethanes.

Supplementary figures

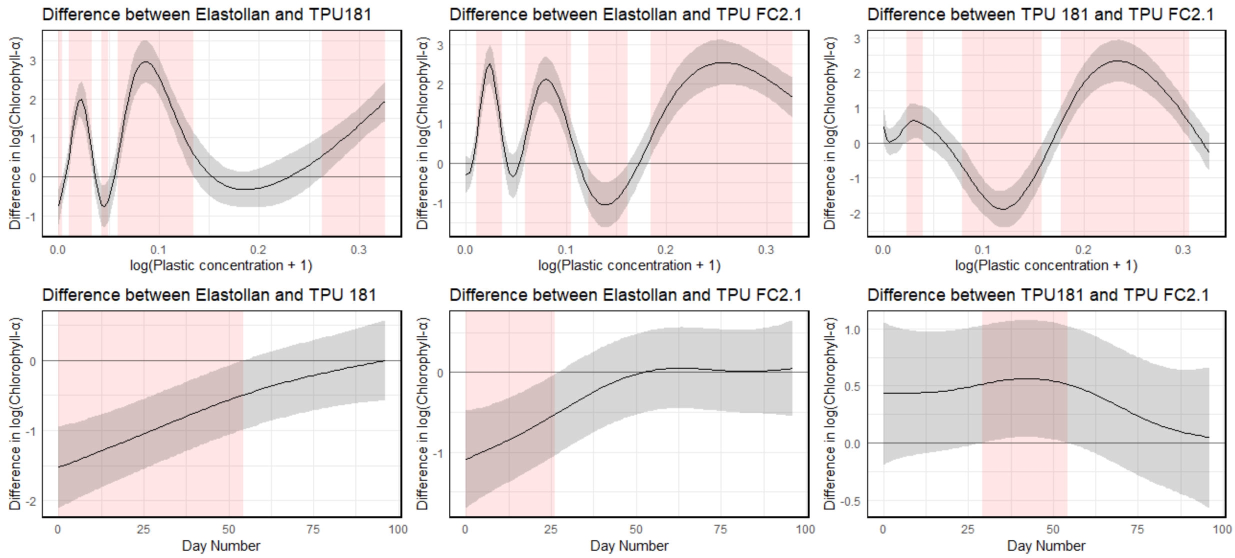

Supplementary Fig. 1 Significant differences in smooth terms for chlorophyll-α: Plot showing pairwise differences in the smooth terms for chlorophyll-α between treatments, based on the best fit generalized additive model (GAM). The top row shows differences across plastic concentration, and the bottom row shows differences across day number. Shaded regions represent 95% confidence intervals. Red shaded areas indicate significant divergence between groups.

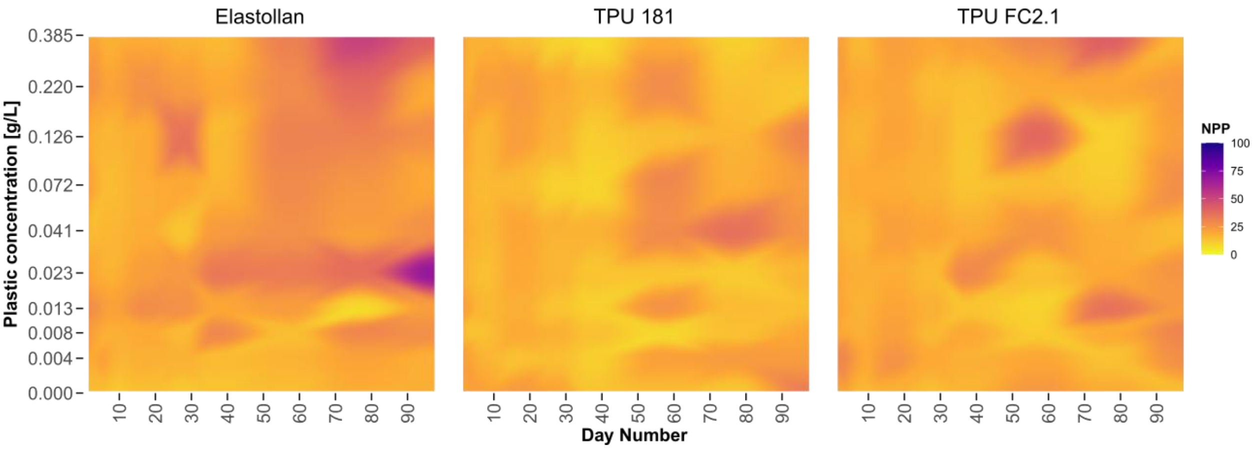

Supplementary Fig. 2 Net primary production: Heatmap of net primary production (NPP) for each plastic type across all plastic concentrations over time. Data were interpolated between the nearest NPP measurements to represent a continuous gradient over time.

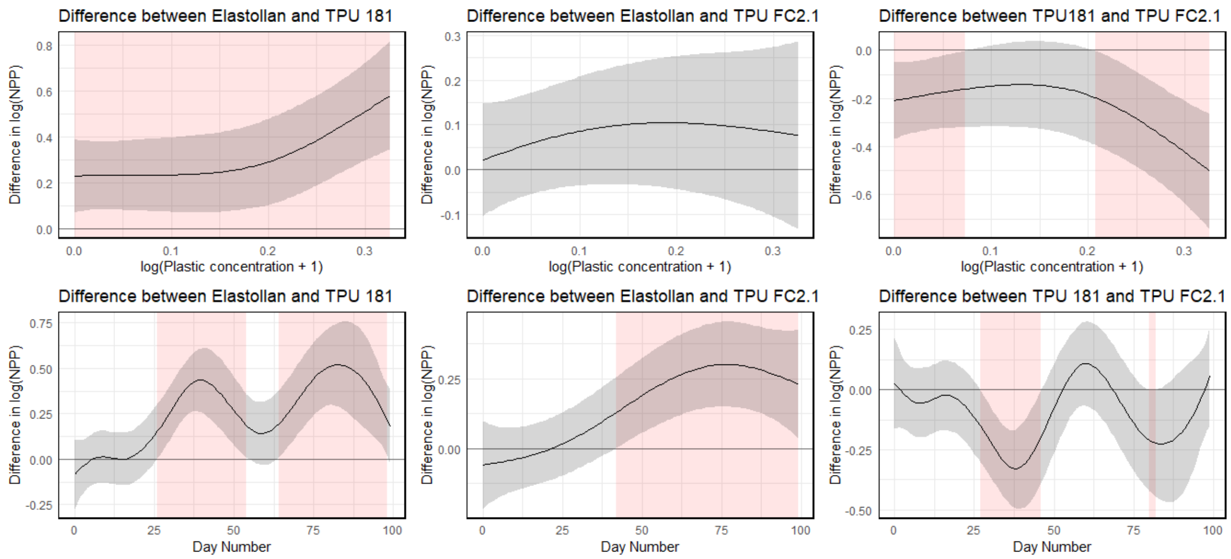

Supplementary Fig. 3 Significant differences in smooth terms for net primary production: Plot showing pairwise differences in the smooth terms for net primary production (NPP) between treatments, based on the best fit generalized additive model (GAM). The top row shows differences across plastic concentration, and the bottom row shows differences across day number. Shaded regions represent 95% confidence intervals. Red shaded areas indicate significant divergence between groups.

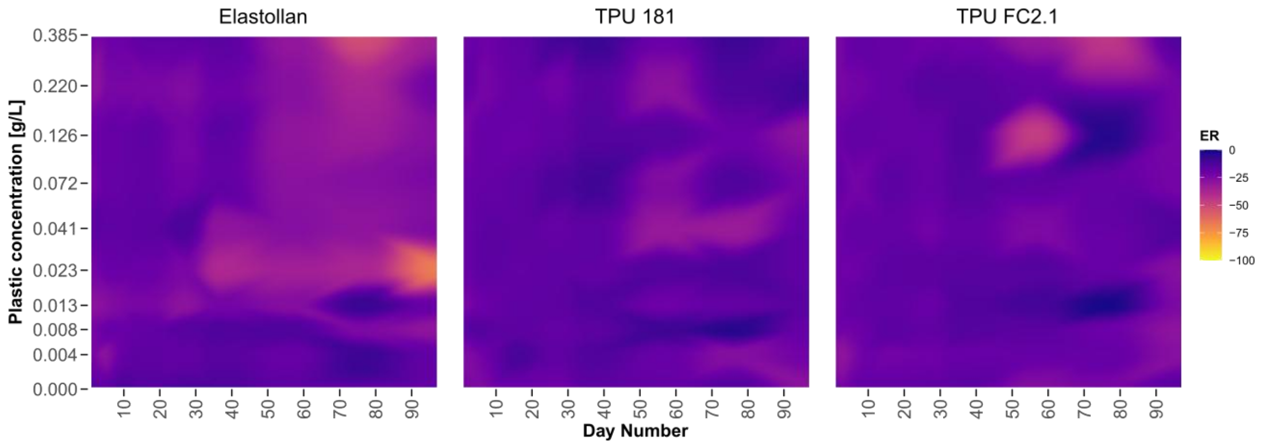

Supplementary Fig. 4 Ecosystem respiration: Heatmap of ecosystem respiration (ER) for each plastic type across all plastic concentrations over time. Data were interpolated between the nearest ER measurements to represent a continuous gradient over time.

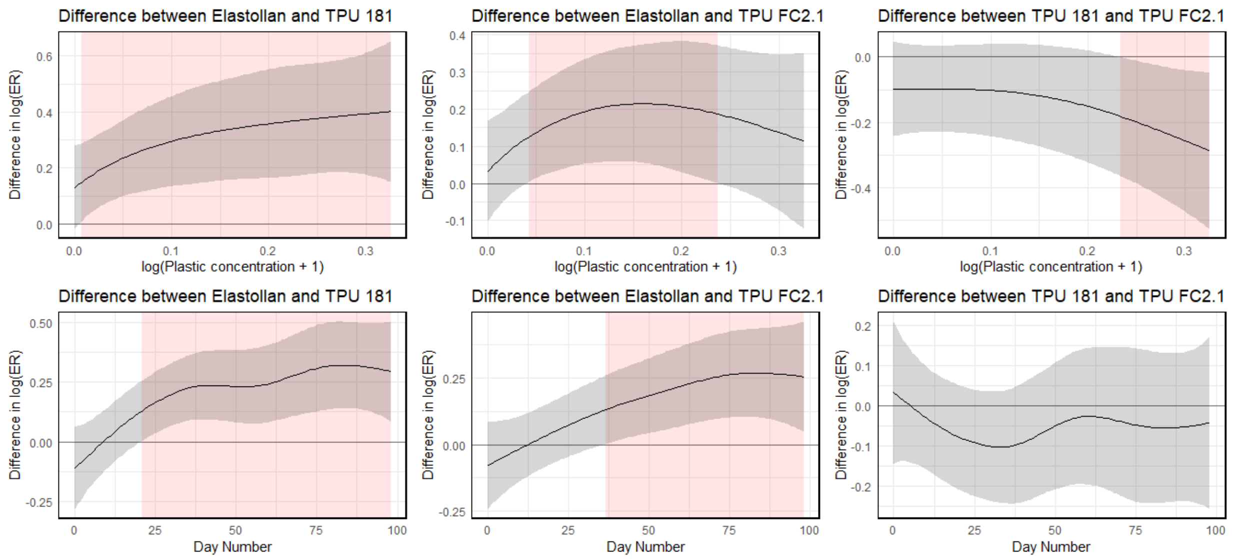

Supplementary Fig. 5 Significant differences in smooth terms for ecosystem respiration: Plot showing pairwise differences in the smooth terms for ecosystem respiration (ER) between treatments, based on the best fit generalized additive model (GAM). The top row shows differences across plastic concentration, and the bottom row shows differences across day number. Shaded regions represent 95% confidence intervals. Red shaded areas indicate significant divergence between groups.

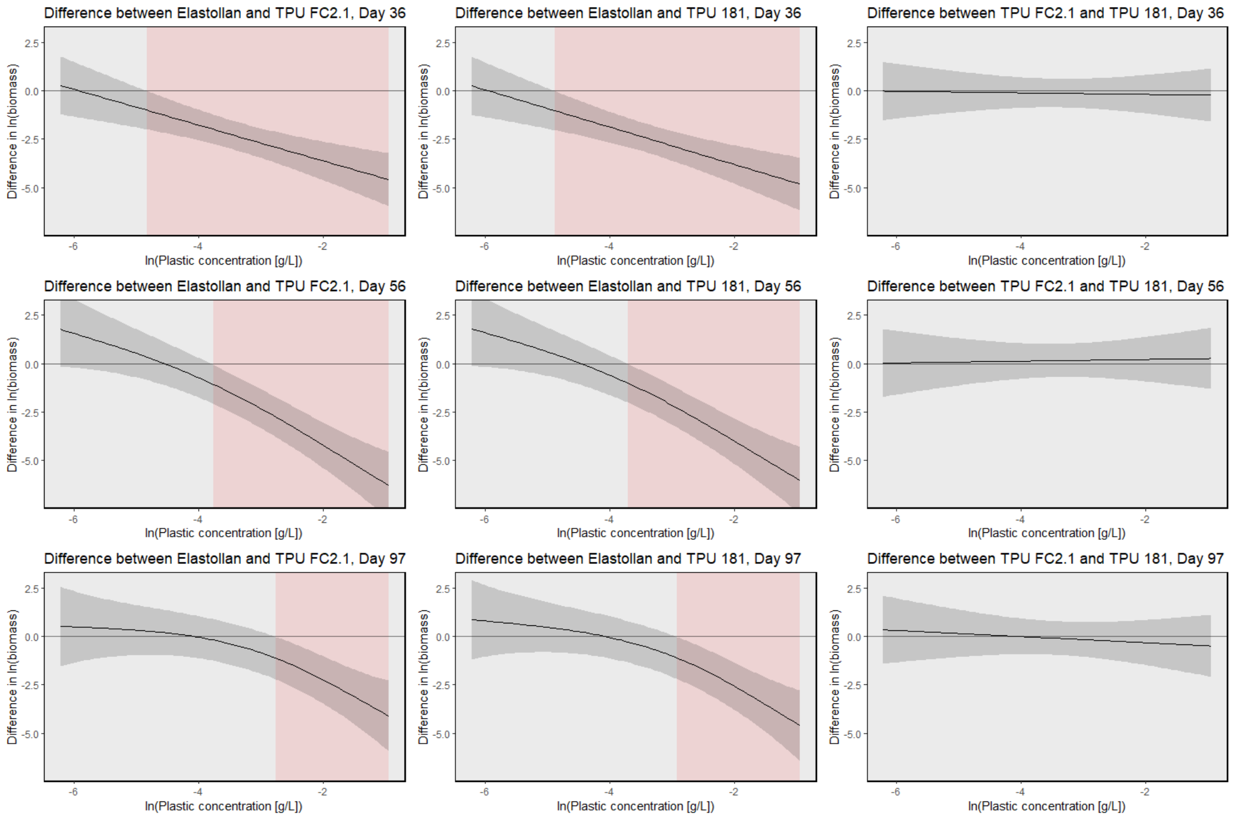

Supplementary Fig. 6 Significant differences in smooth terms for zooplankton biomass: Plot showing pairwise differences in the smooth terms for zooplankton biomass between treatments, based on the best fit generalized additive model (GAM). Each row represents a different sampling date across plastic concentration. Shaded regions represent 95% confidence intervals, and red shaded areas indicate significant divergence between groups.

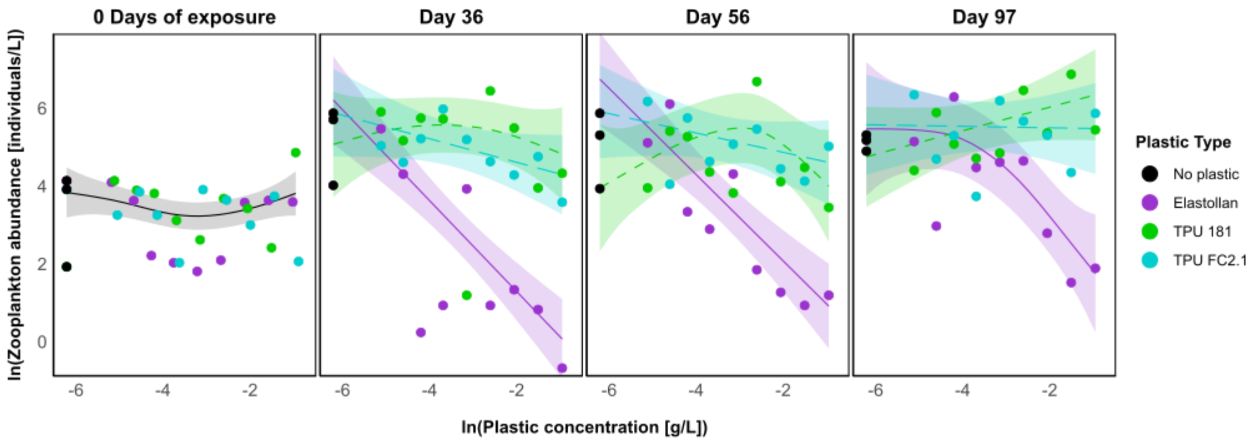

Supplementary Fig. 7 Zooplankton abundance across plastic concentration and exposure times Zooplankton abundance [individuals/liter] by plastic concentration [g/L] for each plastic type before application of treatments (left panel, 0 days of exposure) and for 3 dates after the addition of plastics (3 right panels, day 36, 56, and 97). Lines in each plot represent the estimated value across concentrations with a 95% confidence interval.

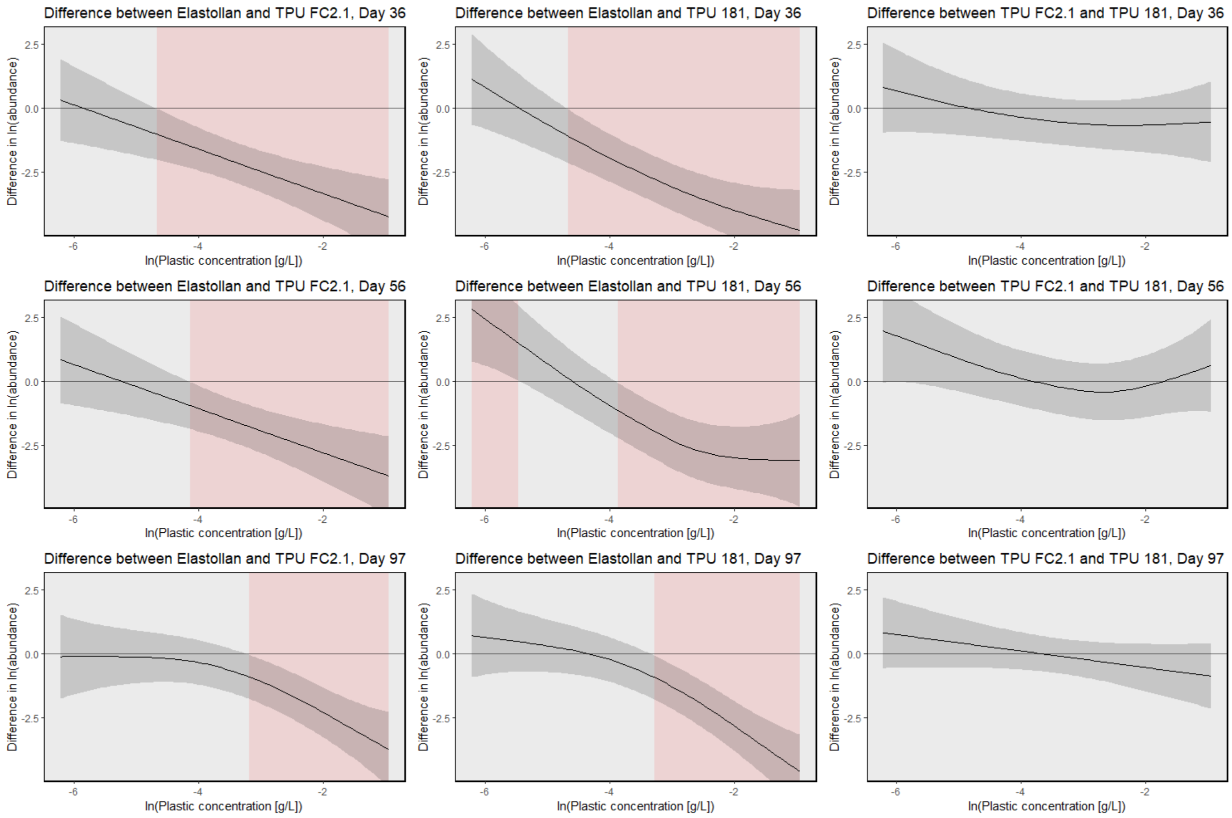

Supplementary Fig. 8 Significant differences in smooth terms for zooplankton abundance: Plot showing pairwise differences in the smooth terms for zooplankton abundance between treatments, based on the best fit generalized additive model (GAM). Each row represents a different sampling date across plastic concentration. Shaded regions represent 95% confidence intervals, and red shaded areas indicate significant divergence between groups.

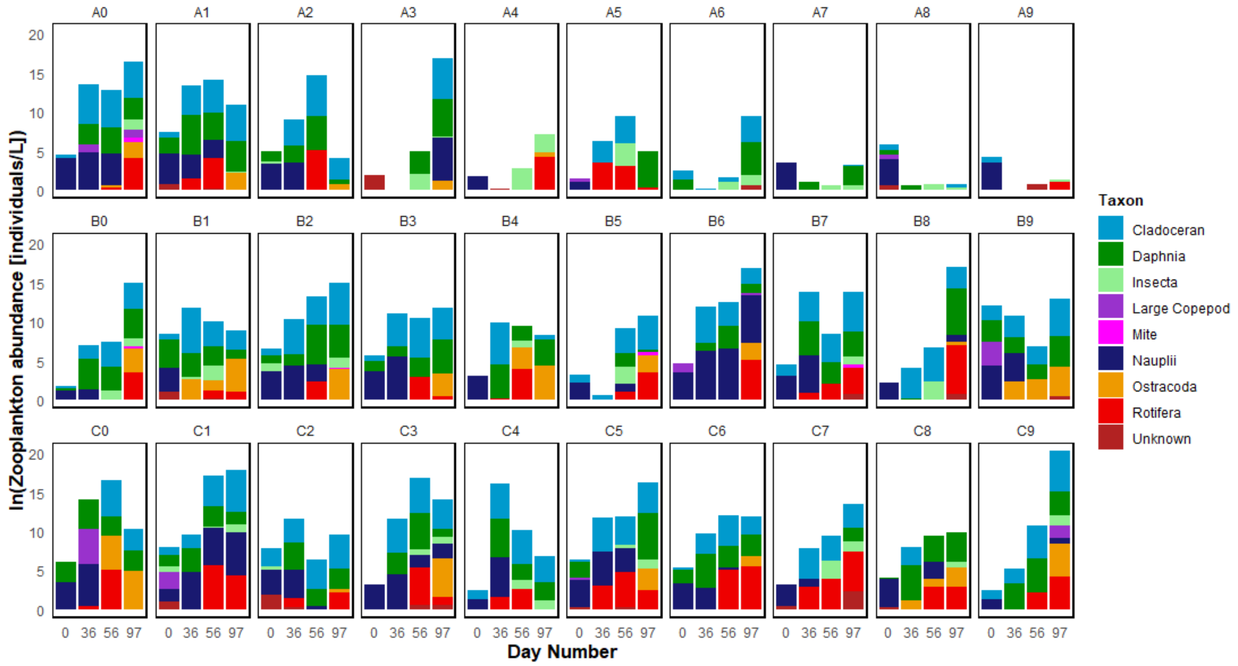

Supplementary Fig. 9 Zooplankton abundance: Natural log-transformed zooplankton abundance (ln[individuals/L]) in all experimental ponds on days 0, 36, 56, and 97. Treatments are labeled as A (Elastollan), B (TPU 181), and C (TPU FC2.1), with numbers 0–9 representing increasing experimental concentrations of microplastics. The specific concentrations (g/L) corresponding to labels 0–9 are as follows: 0 = 0.000, 1 = 0.004, 2 = 0.010, 3 = 0.013, 4 = 0.023, 5 = 0.041, 6 = 0.072, 7 = 0.126, 8 = 0.220, and 9 = 0.385.

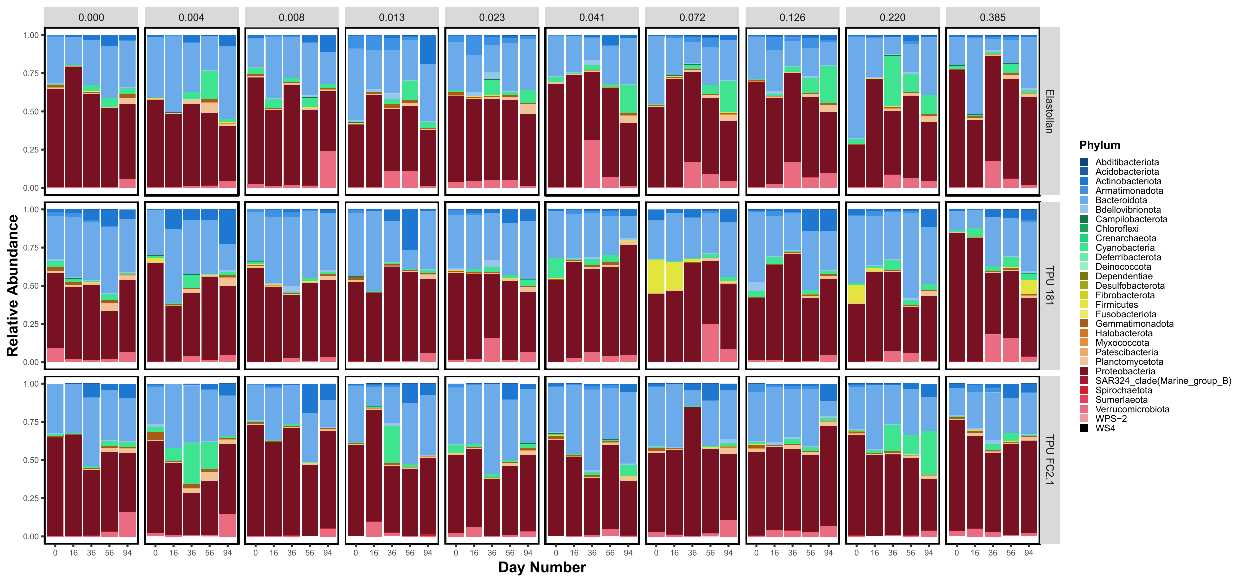

Supplementary Fig. 10 Relative Abundance of 16S Phyla: Relative abundance of 16S phyla in all experimental ponds before the application of plastics (0 days of exposure) and for 4 sampling periods after the addition of plastics (day 16, 36, 56, and 94).

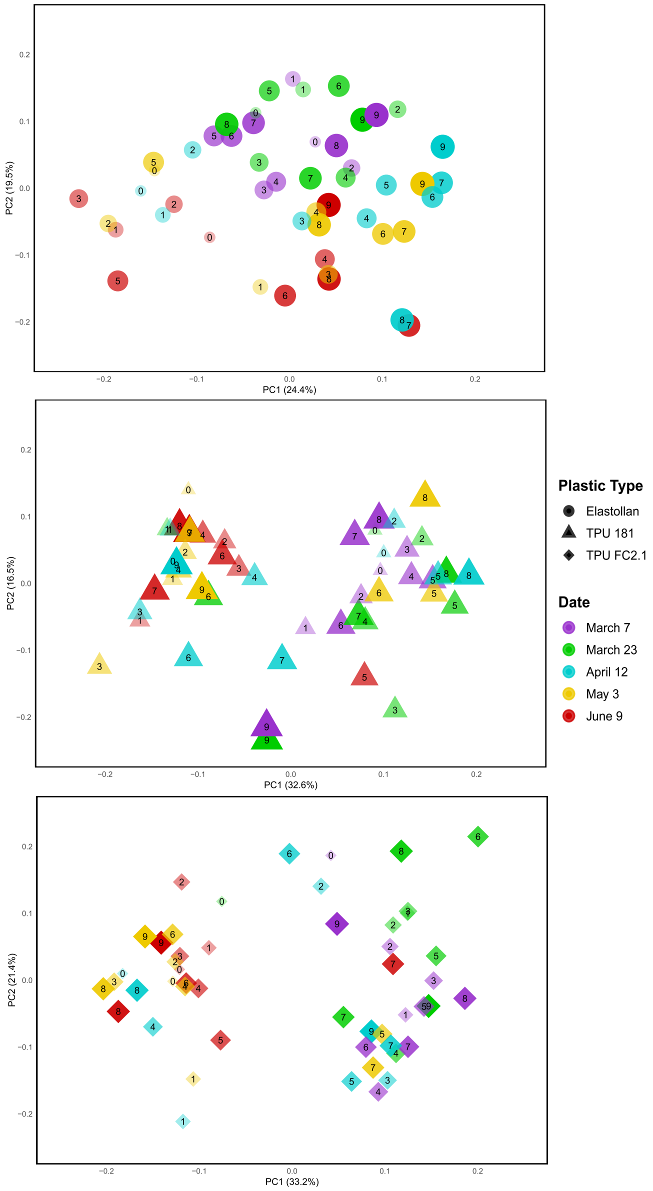

Supplementary Fig. 11 PCoA 16S all dates: Principal Coordinates Analysis (PCoA) plots of 16S rRNA data showing the community composition associated with each plastic type across all sampling time points. Colors indicate different sampling time points, and shapes represent plastic type. Numbers within each symbol denote the 10 unique plastic concentrations, including control (0) tanks.

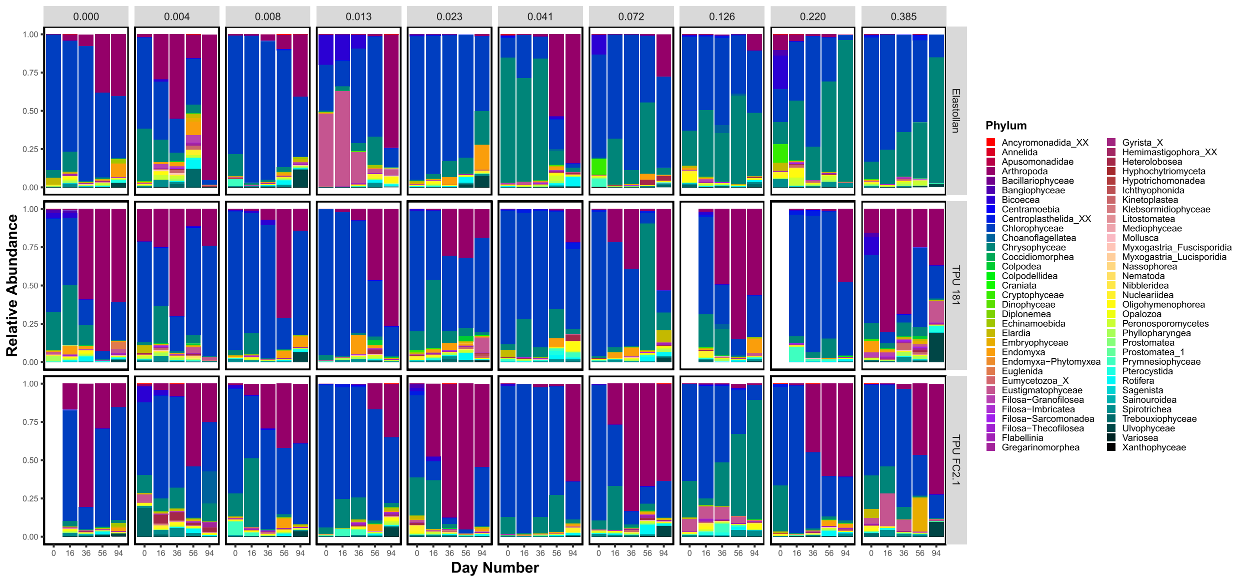

Supplementary Fig. 12 Relative Abundance of 18S Phyla: Relative abundance of eukaryotic phyla in all experimental ponds before the application of plastics (0 days of exposure) and for 4 sampling periods after the addition of plastics (day 16, 36, 56, and 94).

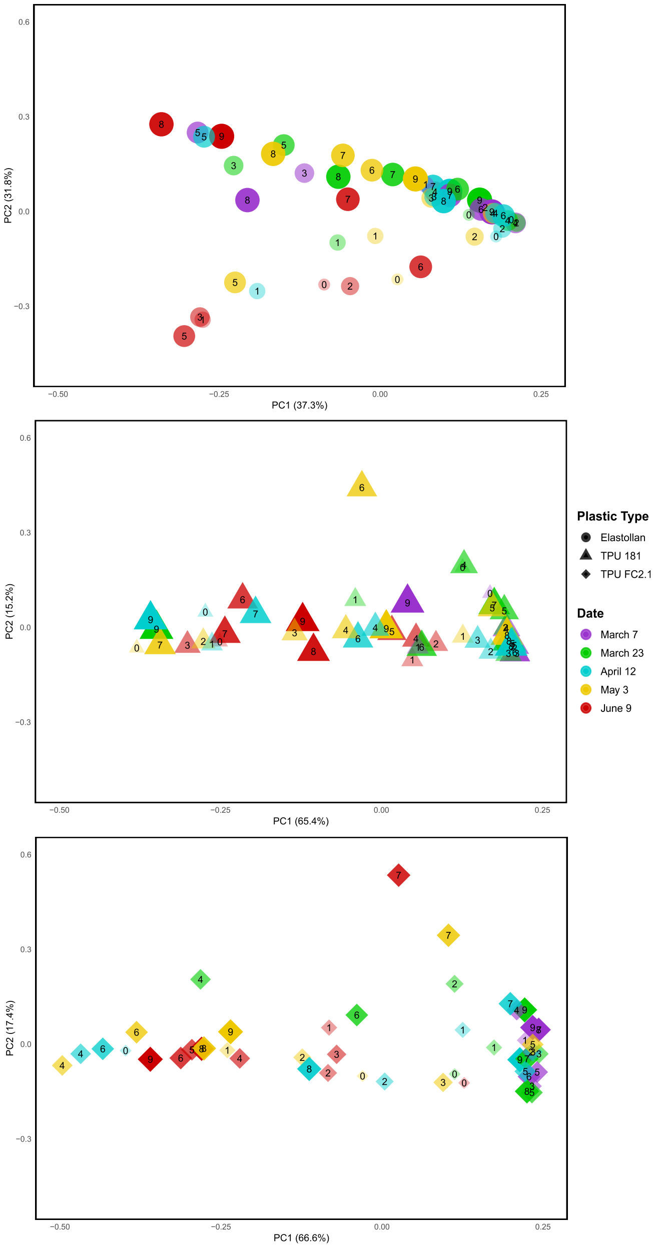

Supplementary Fig. 13 PCoA 18S all dates: Principal Coordinates Analysis (PCoA) plots of 18S rRNA data showing the community composition associated with each plastic type across all sampling time points. Colors indicate different sampling time points, and shapes represent plastic type. Numbers within each symbol denote the 10 unique plastic concentrations, including control tanks.

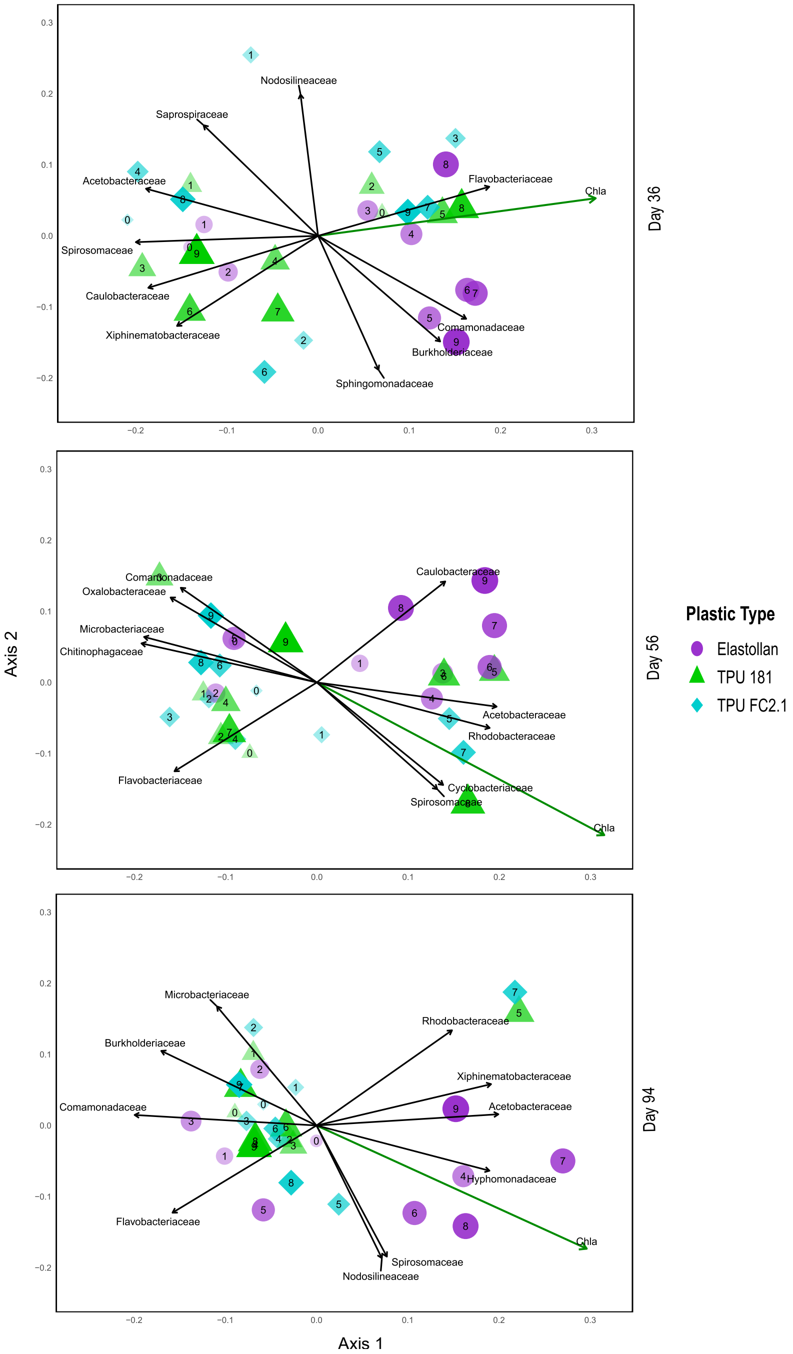

Supplementary Fig. 14 PCoA ordination with biplots of key 16S taxa: Principal coordinates analysis (PCoA) ordination plots with biplots overlaid, showing the ten most abundant bacterial families among those significantly correlated with community composition shifts on days 36, 56, and 94 in the 16S dataset. Taxa are represented as vectors indicating their directional influence on community structure across treatments.

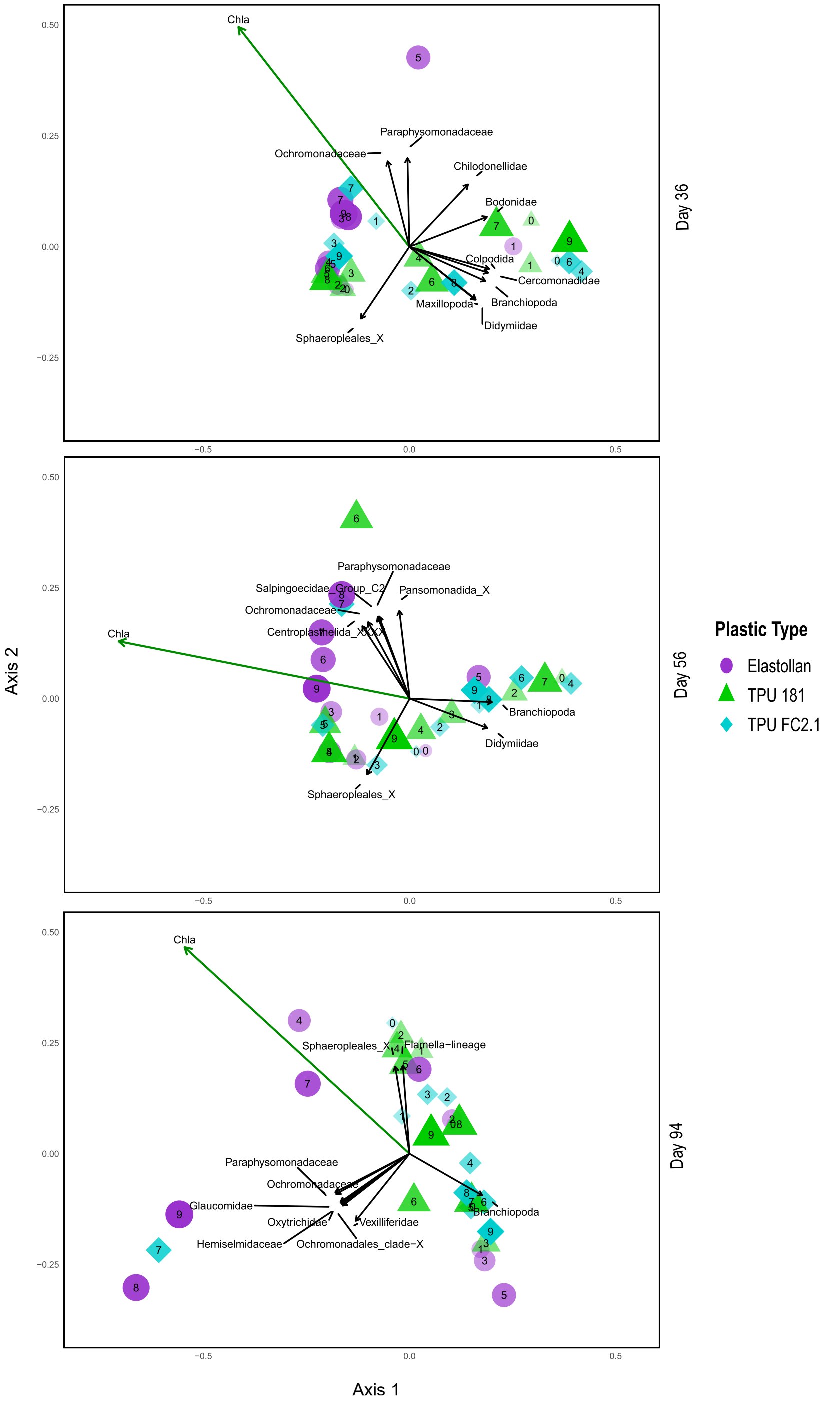

Supplementary Fig. 15 PCoA ordination with biplots of key 18S taxa: Principal coordinates analysis (PCoA) ordination plots with biplots overlaid, showing the ten most abundant bacterial families among those significantly correlated with community composition shifts on days 36, 56, and 94 in the 18S dataset. Taxa are represented as vectors indicating their directional influence on community structure across treatments.

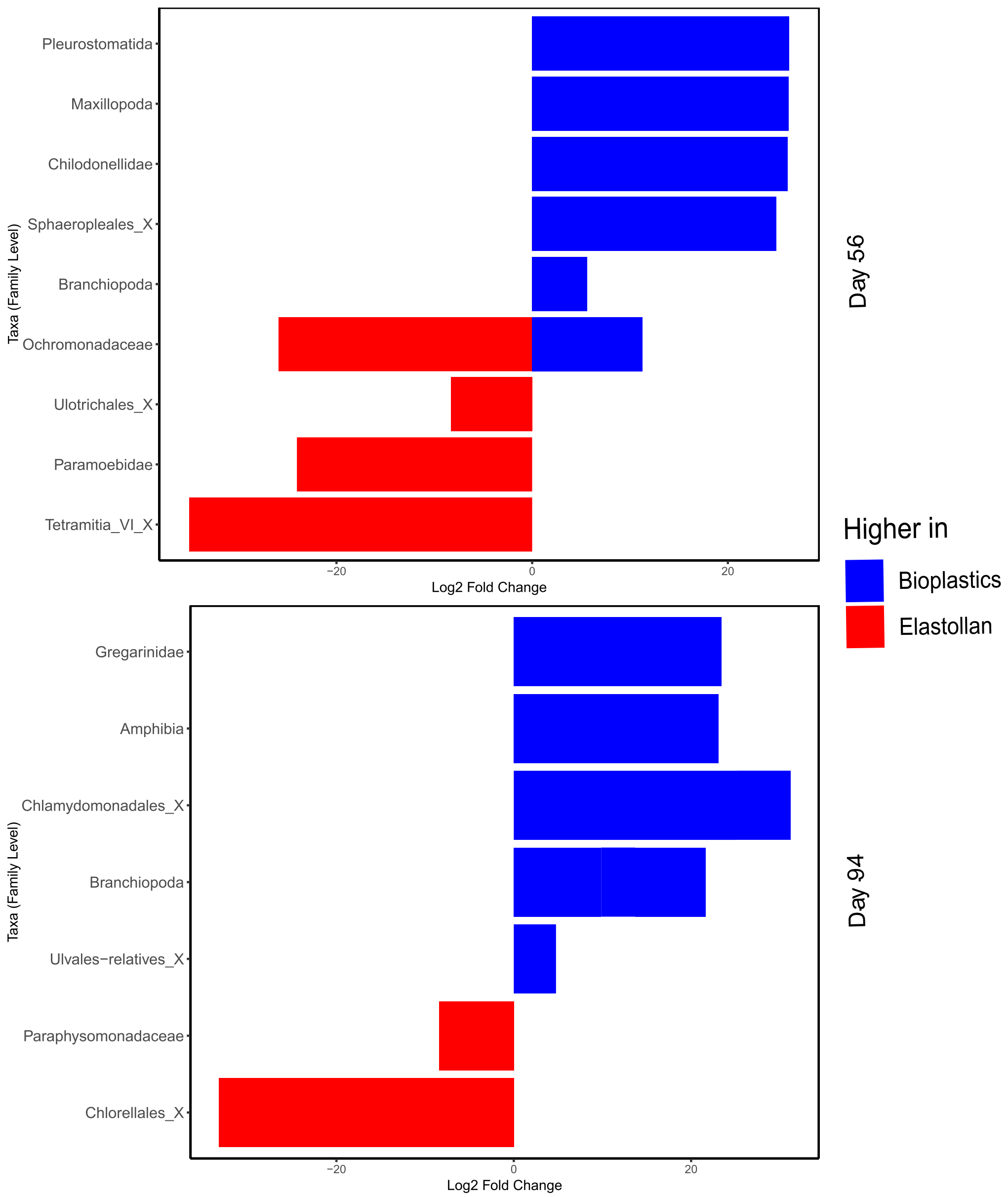

Supplementary Fig. 16 Differential abundance of enriched 18S taxa on day 56 and 94: Barplots showing significantly enriched taxa in the 18S datasets, comparing bioplastic and Elastollan tanks from the six highest plastic concentrations on day 56 (top panel) and day 94 (bottom panel). Bars represent taxa with significant $\log_2$ fold changes (padj < 0.05) normalized to control tanks, highlighting differences in community composition between treatment groups.

Supplementary Table 1 Model selection results for chlorophyll-α, NPP, and ER

Metric	*Model*	*df*	*AIC*	*ΔAIC*	Adjusted R^2	Deviance Explained (%)
**Chlα**	~s(day) + s(log_con)	589.647	1750.777			
	~s(day) + s(log_con) + plastic_type	587.548	1716.672			
	~s(day, by=plastic_type) + s(log_con, by=plastic_type)	571.414	1505.872			
	~s(day, by=plastic_type) + s(log_con, by=plastic_type) + plastic_type	568.524	1446.793	-303.984	0.547	56.9
**NPP**	~s(day) + s(log_con)	10.333	686.415			
	~s(day) + s(log_con) + plastic_type	12.505	660.691			
	~s(day, by=plastic_type) + s(log_con, by=plastic_type)	20.210	675.805			
	~s(day, by=plastic_type) + s(log_con, by=plastic_type) + plastic_type	22.572	648.564	-37.850	0.147	17.0
**ER**	~s(day) + s(log_con)	10.028	361.444			
	~s(day) + s(log_con) + plastic_type	12.210	343.972			
	~s(day, by=plastic_type) + s(log_con, by=plastic_type)	16.135	366.300			
	~s(day, by=plastic_type) + s(log_con, by=plastic_type) + plastic_type	18.492	348.839	-12.605	0.113	14.1

Metric refers to the ecological response variable analyzed (chlorophyll-α, NPP, or ER). *Model* indicates the fixed effects structure of each candidate model. *df* is the number of estimated degrees of freedom. *AIC* is Akaike's Information Criterion value, and *ΔAIC* is the difference in AIC between the simplest model and the selected model. *Adjusted* R^2 represents the proportion of variance explained by the model, adjusted for model complexity. *Deviance Explained (%)* indicates the percentage of deviance in the response variable accounted for by the model.

Supplementary Table 2 Generalized additive model (GAM) results for chlorophyll-α, NPP, and ER across plastic type

Metric	Model	*Estimate*	*Std. Error*	*t-value*	*df/edf*	*Ref.df*	*F*	*p-value*
**Chlα**	~s(day, by=plastic_type) + s(log_con, by=plastic_type)							
	Parametric: Plastic Type Elastollan (*intercept*)	3.187	0.056	57.310				<0.0001
	Parametric: Plastic Type TPU 181	-0.567	0.079	-7.213				<0.0001
	Parametric: Plastic Type TPU FC2.1	-0.509	0.079	-6.482				<0.0001
	s(day) : Elastollan				1.317	1.567	5.982	0.0047
	s(day): TPU 181				1.750	2.181	13.056	<0.0001
	s(day) : TPU FC2.1				2.922	3.629	4.980	0.0019
	s(log_con) : Elastollan				7.171	7.719	28.958	<0.0001
	s(log_con): TPU 181				7.168	7.717	25.806	<0.0001
	s(log_con) : TPU FC2.1				7.147	7.697	24.344	<0.0001
**NPP**	~s(day, by=plastic_type) + s(log_con, by=plastic_type) + plastic_type							
	Parametric: Plastic Type Elastollan (*intercept*)	3.058	0.025	120.283				<0.0001
	Parametric: Plastic Type TPU 181	-0.200	0.036	-5.570				<0.0001
	Parametric: Plastic Type TPU FC2.1	-0.102	0.036	-2.839				0.0046
	s(day) : Elastollan				2.602	3.184	8.447	<0.0001
	s(day): TPU 181				5.907	6.729	6.699	<0.0001
	s(day) : TPU FC2.1				2.428	2.974	1.937	0.1113
	s(log_con) : Elastollan				1.050	1.098	5.206	0.0182
	s(log_con): TPU 181				2.227	2.682	2.017	0.0815
	s(log_con) : TPU FC2.1				1.562	1.904	2.141	0.1768
**ER**	~s(day, by=plastic_type) + s(log_con, by=plastic_type) + plastic_type							
	Parametric: Plastic Type Elastollan (*intercept*)	3.093	0.029	107.664				<0.0001
	Parametric: Plastic Type TPU 181	-0.185	0.041	-4.557				<0.0001
	Parametric: Plastic Type TPU FC2.1	-0.115	0.041	-2.842				0.0047
	s(day) : Elastollan				1.770	2.178	5.692	0.00353
	s(day): TPU 181				3.172	3.857	2.960	0.01833
	s(day) : TPU FC2.1				2.305	2.815	1.854	0.15563
	s(log_con) : Elastollan				2.211	2.663	3.458	0.02933
	s(log_con): TPU 181				1.619	1.976	1.066	0.38306
	s(log_con) : TPU FC2.1				1.001	1.002	0.966	0.32674

Metric refers to the ecological response variable analyzed. *Model* indicates the predictor term (e.g., plastic type or smoothing term). *Estimate* is the estimated effect size for parametric terms. *Std. Error* is the standard error of the estimate. *t-value* is the test statistic for parametric terms. *df/edf* is the degrees of freedom (df) for parametric terms or estimated degrees of freedom (edf) for smooth terms. *Ref.df* is the reference degrees of freedom used to calculate the F-statistic. *F* is the F-statistic for smooth terms. *p-value* indicates the significance of the model term.

Supplementary Table 3 Model selection results for zooplankton biomass and abundances

Metric	*Time*	*Model*	*df*	*AIC*	*Δ AIC*	Adjusted R^2	Deviance Explained (%)
**Biomass**	0 Days	~ s(log.con)	7.80	281.9873	0	0.659	45.4
		~ plastic_type + s(log.con)	9.23	283.8462			
		~ plastic_type + s(log.con, by=plastic_type)	9.43	284.0886			
	Day 36	~ s(log.con)	3.00	381.2262			
		~ plastic_type + s(log.con)	5.00	377.2943			
		~ plastic_type + s(log.con, by=plastic_type)	7.00	374.2129	-7.0133	0.191	40.2
	Day 56	~ s(log.con)	3.00	373.8640			
		~ plastic_type + s(log.con)	5.00	376.0841			
		~ plastic_type + s(log.con, by=plastic_type)	8.06	366.2181	-7.6459	-1.940	43.2
	Day 97	~ s(log.con)	3.00	420.2585			
		~ plastic_type + s(log.con)	5.00	422.8167			
		~ plastic_type + s(log.con, by=plastic_type)	8.55	417.2067	-3.0518	-0.035	34.9
**Abundance**	0 Days	~ s(log.con)	4.83	269.7680	0	0.078	14.5
		~ plastic_type + s(log.con)	6.87	272.4354			
		~ plastic_type + s(log.con, by=plastic_type)	13.29	265.1669			
	Day 36	~ s(log.con)	3.00	357.6869			
		~ plastic_type + s(log.con)	5.00	348.9697			
		~ plastic_type + s(log.con, by=plastic_type)	8.01	340.4372	-17.2497	0.257	58.3
	Day 56	~ s(log.con)	3.00	362.4874			
		~ plastic_type + s(log.con)	5.00	360.5040			
		~ plastic_type + s(log.con, by=plastic_type)	8.93	346.8757	-15.6117	-0.450	60.4
	Day 97	~ s(log.con)	3.00	390.1397			
		~ plastic_type + s(log.con)	5.00	389.9740			
		~ plastic_type + s(log.con, by=plastic_type)	8.70	377.8518	-12.2879	0.098	52.4

Metric refers to the zooplankton response variable analyzed (biomass or abundance). *Time* indicates the sampling period included in the model. *Model* describes the structure of each candidate model. *df* is the number of estimated degrees of freedom. *AIC* is Akaike's Information Criterion value, and *ΔAIC* is the difference in AIC between the simplest model and the selected model. *Adjusted* R^2 represents the proportion of variance explained by the model, adjusted for model complexity. *Deviance Explained (%)* indicates the percentage of deviance in the response variable accounted for by the model.

Supplementary Table 4 Generalized additive model (GAM) results for zooplankton biomass and abundance across sampling periods

Metric	*Time*	*Effect*	*df/edf*	*Ref.df*	*F*	*p-value*
**Biomass**	0 Days	s(log.con)	4.792	5.807	2.918	0.0262
	Day 36	plastic_type + s(log.con, by = plastic_type)	2		26.620	<0.0001
		s(log.con) : Elastollan	1	1	54.150	<0.0001
		s(log.con): TPU 181	1	1	2.670	0.1153
		s(log.con) : TPU FC2.1	1	1	3.510	0.0732
	Day 56	plastic_type + s(log.con, by = plastic_type)	2		12.710	0.0002
		s(log.con) : Elastollan	1.654	2.057	36.595	<0.0001
		s(log.con) : TPU 181	1	1	0.579	0.4550
		s(log.con) : TPU FC2.1	1	1	0.282	0.6010
	Day 97	plastic_type + s(log.con, by = plastic_type)	2		4.166	0.0286
		s(log.con) : Elastollan	2.042	2.545	9.428	0.0007
		s(log.con) : TPU 181	1	1	0.915	0.3487
		s(log.con) : TPU FC2.1	1	1	0.024	0.8788
**Aubundance**	0 Days	s(log.con)	2.273	2.825	1.777	0.2540
	Day 36	plastic_type + s(log.con, by = plastic_type)	2		21.320	<0.0001
		s(log.con) : Elastollan	1	1	42.889	<0.0001
		s(log.con): TPU 181	1.621	2.012	0.734	0.4960
		s(log.con) : TPU FC2.1	1	1	2.881	0.1030
	Day 56	plastic_type + s(log.con, by = plastic_type)	2		7.624	0.0029
		s(log.con) : Elastollan	1.002	1.003	34.055	<0.0001
		s(log.con) : TPU 181	2.358	2.927	1.569	0.2700
		s(log.con) : TPU FC2.1	1	1	1.717	0.2030
	Day 97	plastic_type + s(log.con, by = plastic_type)	2		9.068	0.0013
		s(log.con) : Elastollan	2.173	2.704	10.358	0.0003
		s(log.con) : TPU 181	1	1	3.801	0.0502
		s(log.con) : TPU FC2.1	1	1	0.015	0.9047

Metric refers to the zooplankton response variable analyzed (biomass or abundance). *Time* indicates the sampling period included in the model. *Effect* denotes the model term (parametric or smooth) being evaluated. *df/edf* is the degrees of freedom (df) for parametric terms or estimated degrees of freedom (edf) for smooth terms. *Ref.df* is the reference degrees of freedom used in calculating the F-statistic. *F* is the F-statistic for the effect, and *p-value* indicates the significance of the model term.

Supplementary Table 5 Significant pairwise comparisons of zooplankton biomass between plastic treatments across concentrations and sampling dates

Day Number	*Plastic Concentration (g/L)*	*Comparison*	*Estimate ± SE*	*t-ratio*	*p-value*
Day 36	0.008	Elastollan - TPU 181	-1.293 ± 0.473	-2.736	0.0299
	0.008	Elastollan - TPU 2.1	-1.212 ± 0.473	-2.565	0.0433
	0.013	Elastollan - TPU 181	-1.683 ± 0.422	-3.993	0.0015
	0.013	Elastollan - TPU 2.1	-1.586 ± 0.422	-3.762	0.0027
	0.023	Elastollan - TPU 181	-2.176 ± 0.383	-5.678	<0.0001
	0.023	Elastollan - TPU 2.1	-2.058 ± 0.383	-5.371	<0.0001
	0.041	Elastollan - TPU 181	-2.698 ± 0.382	-7.056	<0.0001
	0.041	Elastollan - TPU 2.1	-2.558 ± 0.382	-6.690	<0.0001
	0.072	Elastollan - TPU 181	-3.221 ± 0.423	-7.613	<0.0001
	0.072	Elastollan - TPU 2.1	-3.059 ± 0.423	-7.231	<0.0001
	0.126	Elastollan - TPU 181	-3.749 ± 0.496	-7.560	<0.0001
	0.126	Elastollan - TPU 2.1	-3.565 ± 0.496	-7.189	<0.0001
	0.220	Elastollan - TPU 181	-4.279 ± 0.589	-7.260	<0.0001
	0.220	Elastollan - TPU 2.1	-4.073 ± 0.589	-6.911	<0.0001
	0.385	Elastollan - TPU 181	-4.814 ± 0.696	-6.916	<0.0001
	0.385	Elastollan - TPU 2.1	-4.586 ± 0.696	-6.588	<0.0001
Day 56	0.041	Elastollan - TPU 181	-1.895 ± 0.509	-3.723	0.0030
	0.041	Elastollan - TPU 2.1	-2.059 '± 0.509	-4.044	0.0014
	0.072	Elastollan - TPU 181	-2.853 ± 0.534	-5.344	0.0001
	0.072	Elastollan - TPU 2.1	-3.041 ± 0.534	-5.696	<0.0001
	0.126	Elastollan - TPU 181	-3.89 ± 0.593	-6.563	<0.0001
	0.126	Elastollan - TPU 2.1	-4.103 ± 0.593	-6.922	<0.0001
	0.220	Elastollan - TPU 181	-4.956 ± 0.703	-7.050	<0.0001
	0.220	Elastollan - TPU 2.1	-5.193 ± 0.703	-7.387	<0.0001
	0.385	Elastollan - TPU 181	-6.036 ± 0.884	-6.824	<0.0001
	0.385	Elastollan - TPU 2.1	-6.298 ± 0.884	-7.120	<0.0001
Day 97	0.072	Elastollan - TPU 181	-1.547 ± 0.568	-2.723	0.0314
	0.126	Elastollan - TPU 181	-2.467 ± 0.616	-4.007	0.0015
	0.126	Elastollan - TPU 2.1	-2.15 ± 0.616	-3.492	0.0054
	0.220	Elastollan - TPU 181	-3.512 ± 0.719	-4.882	0.0002
	0.220	Elastollan - TPU 2.1	-3.107 ± 0.719	-4.320	0.0007
	0.385	Elastollan - TPU 181	-4.605 ± 0.931	-4.949	0.0002
	0.385	Elastollan - TPU 2.1	-4.113 ± 0.931	-4.420	0.0006

Day Number indicates the sampling period. *Plastic Concentration (g/L)* refers to the concentration of plastic associated with each comparison. *Comparison* specifies the pair of plastic treatments being compared. *Estimate ± SE* is the difference in estimated zooplankton biomass between the

two treatments, reported with the standard error. *t-ratio* is the test statistic from the pairwise comparison. *p-value* indicates the significance of the comparison.

Supplementary Table 6 16S and 18S global PERMANOVA results

Metric	*Effect*	*df*	*SumofSqs*	R^2	*F*	*p-value*
**16S**	plastic_type	2	0.2032	0.02998	2.8401	0.00400
	plastic_concentration	1	0.0780	0.01151	2.1807	0.02997
	date	4	1.0643	0.15706	7.4384	0.00100
	chlα	1	0.3345	0.04936	9.3510	0.00100
	plastic_type:plastic_concentration	2	0.151	0.22280	2.1108	0.01399
	plastic_type:date	8	0.3638	0.05368	1.2712	0.09391
	plastic_concentration:date	4	0.1072	0.15820	0.7493	0.85315
	plastic_type:plastic_concentration_date	8	0.2178	0.03214	0.7611	0.92408
**18S**	plastic_type	2	0.3852	0.04204	4.1079	0.00300
	plastic_concentration	1	0.0897	0.00979	1.9141	0.11688
	date	4	1.5304	0.16703	8.1611	0.00100
	chlα	1	0.4952	0.05405	10.5625	0.00100
	plastic_type:plastic_concentration	2	0.2396	0.02615	2.5551	0.03497
	plastic_type:date	8	0.3617	0.03948	0.9645	0.50350
	plastic_concentration:date	4	0.1797	0.01962	0.9584	0.46154
	plastic_type:plastic_concentration_date	8	0.4427	0.04832	1.1803	0.25475

Metric refers to the dataset analyzed (16S or 18S). *Effect* indicates the explanatory variable tested. *df* is the degrees of freedom for each effect. *SumofSqs* is the sum of squares associated with the effect. R^2 represents the proportion of variance explained by the effect. *F* is the F-statistic from the PERMANOVA test. *p-value* indicates the significance of the effect.

Supplementary Table 7 Date specific 16S and 18S PERMANOVA results

Metric	*Time*	*Effect*	*df*	*SumofSqs*	*R^2*	*F*	*p-value*
**16S**	0 Days	plastic_type	2	0.0813	0.0848	1.2438	0.1848
		plastic_concentration	1	0.0374	0.0391	1.1455	0.2967
		chla	1	0.4486	0.0468	1.3729	0.2058
		plastic_type:plastic_concentration	2	0.0434	0.0453	0.6643	0.8561
	Day 16	plastic_type	2	0.1167	0.1037	1.6519	0.0839
		plastic_concentration	1	0.0168	0.0149	0.4757	0.8801
		chla	1	0.1105	0.0982	3.1284	0.0060
		plastic_type:plastic_concentration	2	0.0685	0.0609	0.9705	0.4525
	Day 36	plastic_type	2	0.1523	0.1111	1.9516	0.0370
		plastic_concentration	1	0.0533	0.0389	1.3658	0.2068
		chla	1	0.1846	0.1347	4.7313	0.0010
		plastic_type:plastic_concentration	2	0.0833	0.0608	1.0673	0.4086
	Day 56	plastic_type	2	0.1388	0.1179	2.3266	0.0220
		plastic_concentration	1	0.0538	0.0455	1.8030	0.0969
		chla	1	0.2077	0.1764	6.9608	0.0010
		plastic_type:plastic_concentration	2	0.0910	0.0773	1.5253	0.1319
	Day 94	plastic_type	2	0.1104	0.0964	1.4821	0.1039
		plastic_concentration	1	0.0271	0.0251	0.7717	0.6134
		chla	1	0.0570	0.0527	1.6205	0.1439
		plastic_type:plastic_concentration	2	0.0846	0.0783	1.2036	0.2627
**18S**	0 Days	plastic_type	2	0.0842	0.0976	1.3657	0.2058
		plastic_concentration	1	0.0383	0.0444	1.2421	0.2797
		chla	1	0.0730	0.0846	2.3678	0.0799
		plastic_type:plastic_concentration	2	0.0510	0.0591	0.8270	0.4565
	Day 16	plastic_type	2	0.0510	0.0379	0.5947	0.7892
		plastic_concentration	1	0.0138	0.0102	0.3208	0.8621
		chla	1	0.1263	0.0939	2.9455	0.0340
		plastic_type:plastic_concentration	2	0.1671	0.1244	1.9495	0.1079
	Day 36	plastic_type	2	0.1980	0.1131	1.9187	0.1019
		plastic_concentration	1	0.0233	0.0133	0.4524	0.6444
		chla	1	0.2478	0.1415	4.8023	0.0070
		plastic_type:plastic_concentration	2	0.0956	0.0546	0.9264	0.4565
	Day 56	plastic_type	2	0.2483	0.1338	2.2851	0.0849
		plastic_concentration	1	0.0543	0.0293	1.0003	0.3676
		chla	1	0.1998	0.1077	3.6781	0.0170
		plastic_type:plastic_concentration	2	0.1037	0.0559	0.9539	0.4266
	Day 94	plastic_type	2	0.1176	0.6539	1.2036	0.2917
		plastic_concentration	1	0.1393	0.0775	2.8528	0.0410
		chla	1	0.1490	0.0829	3.0512	0.0739
		plastic_type:plastic_concentration	2	0.2689	0.1495	2.7526	0.0320

Metric refers to the dataset analyzed (16S or 18S). *Time* indicates the specific sampling period on which the analysis was conducted. *Effect* is the explanatory variable tested (e.g., plastic type, concentration). *df* is the degrees of freedom associated with each effect. *SumofSqs* is the sum of squares attributed to the effect. R^2 represents the proportion of variance in community composition explained by the effect. *F* is the F-statistic from the PERMANOVA test. *p-value* indicates the significance of the effect on that date.

Supplementary Table 8 Community metrics for no-plastic control tanks.

Dataset	R^2	*F*	*p*
Zoop Abundance			
PERMANOVA	0.270	1.666	0.095
ANOVA		2.511	0.136
Shannon		0.816	0.472
16S			
PERMANOVA	0.240	1.898	0.053
ANOVA		1.000	0.397
Shannon		0.290	0.753
18S			
PERMANOVA	0.200	1.421	0.250
ANOVA		0.411	0.673
Shannon		0.855	0.452

Results show variation over time in control tanks based on PERMANOVA (community composition), ANOVA on total abundance and Shannon diversity. R^2, F-statistics, and p-values are reported for each test. These analyses assess background temporal variation in the absence of plastic exposure.

Supplementary Table 9 Water quality summary for all ponds on day 0

Variable	*Min*	*Mean*	*SD*	*Max*
Temperature (°C)	12.2	12.443	0.104	12.6
Dissolved O_2 (%)	88.3	100.763	5.273	113.9
Dissolved O_2 (mg/L)	9.37	10.726	0.567	12.22
Conductivity (μS)	0.64	0.672	0.024	0.754
TDS (g/L)	0.416	0.437	0.015	0.49
Salinity (ppt)	0.31	0.329	0.013	0.37
pH	9.2	9.433	0.152	9.78
Chlorophyll-a (μg/L)	9.732	29.985	15.907	71.562

Min, mean, standard deviation (SD), and maximum values are shown for each variable based on measurements collected from all ponds prior to the addition of plastics.
