# Supplementary material for: Microplastic pollution induces algae blooms in experimental ponds but bioplastics are less harmful
Source: Commun Sustain. 2026 Jan 19;1(1):16. doi: 10.1038/s44458-025-00014-6 (PMC12863645; doi:10.1038/s44458-025-00014-6)
Supplement: Supplementary file 1 — Transparent Peer Review file [file 44458_2025_14_MOESM1_ESM.pdf]

nature portfolio

Peer Review File

Microplastic pollution induces algae blooms in experimental ponds but bioplastics are less harmful

Corresponding Author: Mr Scott Morton

This file contains all editorial decision letters in order by version, followed by all author rebuttals in order by version.

Attachments originally included by the reviewers as part of their assessment can be found at the end of this file.

Version 0:

Decision Letter:

** Please ensure you delete the link to your author home page in this e-mail if you wish to forward it to your coauthors **

Dear Mr Morton,

Your manuscript titled "Microplastic pollution induces algae blooms in experimental ponds but bioplastics are less harmful" has now been seen by 2 reviewers, and we include their comments at the end of this message. They find your work of interest, but some important points are raised. We are interested in the possibility of publishing your study in Communications Sustainability, but would like to consider your responses to these concerns and assess a revised manuscript before we make a final decision on publication.

Please ensure that your methodology is fully justified and described at a level of detail that will allow independent researchers to replicate your results.

We therefore invite you to revise and resubmit your manuscript, along with a point-by-point response that takes into account the points raised. Please highlight all changes in the manuscript text file.

Please submit your point-by-point responses as a separate file, distinct from your cover letter where you can add responses to the Editors' comments that you do not want to be made available to the reviewers. Word files are preferred. We recommend that any figures, tables or graphs that are included in the response to reviewers are also included in the main article or Supplementary Information.

We are committed to providing a fair and constructive peer-review process. Please don't hesitate to contact us if you wish to discuss the revision in more detail.

Please use the following link to submit your revised manuscript, point-by-point response to the referees' comments (which should be in a separate document to any cover letter), a tracked-changes version of the manuscript (as a PDF file) and the completed checklist:
Link Redacted
** This url links to your confidential home page and associated information about manuscripts you may have submitted or be reviewing for us. If you wish to forward this email to co-authors, please delete the link to your homepage first **

We hope to receive your revised paper within six weeks; please let us know if you aren't able to submit it within this time so that we can discuss how best to proceed. If we don't hear from you, and the revision process takes significantly longer, we may close your file. In this event, we will still be happy to reconsider your paper at a later date, as long as nothing similar has been accepted for publication at Communications Sustainability or published elsewhere in the meantime.

Please do not hesitate to contact us if you have any questions or would like to discuss these revisions further. We look forward to seeing the revised manuscript and thank you for the opportunity to review your work.

Best regards,

Nandita Basu, PhD
Associate Editor, Communications Sustainability
Consulting Editor, Communications Earth & Environment
Nature Portfolio

EDITORIAL POLICIES AND FORMATTING

We ask that you ensure your manuscript complies with our editorial policies. Please ensure that the following formatting requirements are met, and any checklist relevant to your research is completed and uploaded as a Related Manuscript file type with the revised article.

For Manuscripts that fall into the following fields:
• Behavioural and social science
• Ecological, evolutionary & environmental sciences
• Life sciences
An updated and completed version of our Reporting Summary must be uploaded with the revised manuscript
You can download the form here:
https://www.nature.com/documents/nr-reporting-summary.pdf

Furthermore, please align your manuscript with our format requirements, which are summarized on the following checklist:
Communications Sustainability formatting checklist

and also in our style and formatting guide Communications Sustainability formatting guide .

*** DATA: Communications Sustainability endorses the principles of the Enabling FAIR data project (http://www.copdess.org/enabling-fair-data-project/). We ask authors to make the data that support their conclusions available in permanent, publicly accessible data repositories. (Please contact the editor if you are unable to make your data available).

All Communications Sustainability manuscripts must include a section titled "Data Availability" at the end of the Methods section or main text (if no Methods). More information on this policy, is available at http://www.nature.com/authors/policies/data/data-availability-statements-data-citations.pdf.

In particular, the Data availability statement should include:
- Unique identifiers (such as DOIs and hyperlinks for datasets in public repositories)
- Accession codes where appropriate
- If applicable, a statement regarding data available with restrictions
- If a dataset has a Digital Object Identifier (DOI) as its unique identifier, we strongly encourage including this in the Reference list and citing the dataset in the Data Availability Statement.

DATA SOURCES: All new data associated with the paper should be placed in a persistent repository where they can be freely and enduringly accessed. We recommend submitting the data to discipline-specific, community-recognized repositories, where possible and a list of recommended repositories is provided at http://www.nature.com/sdata/policies/repositories.

If a community resource is unavailable, data can be submitted to generalist repositories such as figshare or Dryad Digital Repository. Please provide a unique identifier for the data (for example a DOI or a permanent URL) in the data availability statement, if possible. If the repository does not provide identifiers, we encourage authors to supply the search terms that will return the data. For data that have been obtained from publicly available sources, please provide a URL and the specific data product name in the data availability statement. Data with a DOI should be further cited in the methods reference section.

Please refer to our data policies at http://www.nature.com/authors/policies/availability.html.

REVIEWER COMMENTS:

Reviewer #1 (Remarks to the Author):

This paper examines the effects of three types of microplastics on planktonic communities. This work is interesting and has certain practical significance. The data and figures in the paper are very rich. However, it is regrettable that the mining of information from these figures is still not sufficient, especially in the discussion section. Therefore, I suggest that the paper needs further major revisions. Here are the detailed comments:

Line 19-20: What kind of response changes? This should be elaborated in detail.

Line 25: Replace "documented" with "detected."

Line 26: Rewrote "some of earth's most remote."

Line 44: "particularly in freshwater ecosystems, but see.?" This does not seem to be a complete sentence.

Line 52: The first mention of TPU should include its full name. And why were TPU 181 and TPU FC2.1 chosen as materials?

Line 71: (Est.=3.451)

Line 75: "Higher concentrations" refers to what level? In Figure 1, it can be observed that in the two biodegradable plastic treatment groups, when the concentration of microplastics is greater than 0.023 g/L, the concentration of chlorophyll a also shows high values.

Line 84-90: You repeatedly mention "lower" and "higher." However, this is a qualitative description. It should be noted that you are describing the results, so you need to provide some specific numbers instead of just describing whether they are lower or higher.

Line 150: Rewrite "in the upper half of the plastic concentration range."

Line 227: Why is it 58:25:17?

Line 228: Since the experimental water came from municipal water, were the background values of the water body in the municipal water measured? For example, concentrations of nitrogen, phosphorus, and chlorophyll a, or environmental parameters (temperature, DO, pH, conductivity, TDS, and salinity), because the nutritional status of these waters can affect plankton.

Line 231-232: Why were different amounts added at different times?

Line 250, 257: I noticed that the sampling times for phytoplankton and microorganisms are not consistent. Why?

The data and figures in the paper are very rich. However, the discussion lacks in-depth analysis. For example, your figures show rich information about microorganisms. However, it is regrettable that the discussion does not cover these results, and thus lacks some eye-catching content for readers. What you have discussed are more general findings. I suggest that the discussion section needs to be further explored in combination with your results.

The clarity of Figure 3 is very poor, and the font is also too small to obtain information. The same applies to Extended Data Figures 1, 2, 6, and 8. Additionally, consider adding 95% confidence ellipses for each treatment group. The distinction between these three colors is not significant enough, and I suggest changing the colors.

Line 863: "with numbers 0–9 indicating increasing experimental concentrations of microplastics." The nine concentrations should be specified.

Reviewer #2 (Remarks to the Author):

The authors have conducted a thorough study that investigates the response of different trophic levels to marine debris plastics. Often sequencing is only on the bacterial level in this field, but a strength of this paper is that they considered algae and protists. They also compared a bioplastic to a petroleum-based plastic in the same category. Some weaknesses of the paper include some missing methodological detail especially around volume, shape and growth conditions of the ponds and some plastic properties. It is unclear if no plastic controls were conducted and/or considered in the statistical analyses, and it is also unclear if replicates were conducted for each concentration. Also, some main figures, extended data and supplementary materials do not have a call-out in the manuscript which required some guessing of what data is the evidence for certain statements. Further some things are not defined at their first mention like what the different plastics actually are or what model 3 is (all in methods which is after intro and results).
Introduction section:
- Third paragraph, last sentence is incomplete.
Results section:
- Chl-a: Figure 1 call-out is missing. Further, it is very confusing to the reader when the first sentence of the results section is a stats result but does not provide context about what experiment was done, what was observed or what results that stats are analyzing.
- Biomass: Did they observe any biofilm formation that might affect biomass readings or are the ponds so big and the concentration of plastics so small that the amount of biomass on the plastic is likely to be negligible?
- Microbial Community: The results section often reports a statistical finding without putting it in the biological context. For

instance, ‘plastic concentration was significant in the 16S dataset but not 18S.’ What was observed to change in the communities with plastic concentration? Does this include a no-plastic control?
Discussion Section:
- I would recommend included a schematic that describes the layers of interactions they think are happening. I agree with the interpretation but a visualization of the trophic levels and differential effect of plastics would be helpful.
- In the second paragraph they describe HABs as unpredictable but in the next sentence they say that they found things that always happen, that phrasing felt contradictory.
- In the third paragraph a discussion of how the no plastic control behaved would help to interpret the acute exposures. Toxicity in these cases is often from the water soluble additives in commercial plastics leaching and disrupting the community. Then once those are consumed, it is more the surface interactions with plastics or ingestion if the pieces are small enough relative to the organism.
Materials and Methods Section/throughout:
- Controls: In some figures it looks like a no plastic control was run, in other figures it is absent. It also unclear if included in stats. It is very important to compare to a no plastic exposure condition. A table or diagram of the experimental set up and all conditions tested would be very helpful.
- Ponds: More information about the ponds/mesocosms (called both in manuscript) must be given. What is their volume and shape? What are they made out of? Are they outside? How is light controlled? Are they stirred, at what rate? Temperature etc? Freshwater, brackish or salt water? Were the pond conditions arranged in a randomized design?
- Replicates: Were replicates conducted or how do they know the observed phenotypes that have many stats run on them are reproducible or reliable?
- Polymer properties of 3 plastics chosen and justification for why these 3 of the thousands of plastics: Please provide a justification for why these 3 plastics out of the thousands of polymers that exist and pollute waterways. Why did the choose two different bioplastics and what are different about these? Do all 3 plastics have similar size, shape, thickness, crystallinity, surface roughness, density etc? We and others globally have found that interactions are very polymer and microbe specific. In order to compare two plastics of bioplastic vs conventional, they should be as similar as possible apart from the origin. The authors also speak about how these plastics will or won’t break down but the plastics are not recovered or tested in this paper limiting their ability to comment on if that was a factor in the observed phenotypes.
- Location of the plastics in the tank and sampling depth and times: These plastics are more dense than water- Do they sink in these tanks? Is there stirring? Are the plastics in the same part of the water column with the algae? How deep into the tanks is the sampling occurring? Was sampling done at the same time of day to control for diurnal variability in community composition?
- Zooplankton identification: no reference is given for ‘available published keys’
- Statistical analyses: If there are no replicates, some explanation should be given for using the concentration gradient as pseudo-replicates or how the stats and models they chose are still appropriate to use without biological replicates. If at least a different no-plastic pond was run for each plastic, did each of the no-plastic ponds behave similarly?
- For all of the Data Tables reporting stats, please define the column headings in footnotes at least once. Also they say model selection but I don’t see the different models represented in the charts. Are the ones displayed model 3 only?
- It wasn’t clear if extended data figures 1 & 2 were called out in the text or what they add beyond what is described in Figure 3.
- For extended data figure 3: Are these normalized to no-plastic?
- Synthesis of TPU 181: methods are missing source of algae-based polyester-polyoyl. Also it states the mechanical properties were tested but not reported nor compared to the other plastics.
- Please make sure all supplementary figures are called-out in the text.

** Visit Nature Portfolio's author and referees' website at www.nature.com/authors for information about policies, services and author benefits**

Communications Sustainability is committed to improving transparency in authorship. As part of our efforts in this direction, we are now requesting that all authors identified as ‘corresponding author’ create and link their Open Researcher and Contributor Identifier (ORCID) with their account on the Manuscript Tracking System prior to acceptance. ORCID helps the scientific community achieve unambiguous attribution of all scholarly contributions. You can create and link your ORCID from the home page of the Manuscript Tracking System by clicking on ‘Modify my Springer Nature account’ and following the instructions in the link below. Please also inform all co-authors that they can add their ORCIDs to their accounts and that they must do so prior to acceptance.
https://www.springernature.com/gp/researchers/orcid/orcid-for-nature-research

For more information please visit http://www.springernature.com/orcid

If you experience problems in linking your ORCID, please contact the Platform Support Helpdesk.

Version 1:

Decision Letter:

<*** REMEMBER TO ATTACH REVISIONS CHECKLIST (WORD)***>

** Please ensure you delete the link to your author home page in this e-mail if you wish to forward it to your coauthors **

Dear Mr Morton,

Your manuscript titled "Microplastic pollution induces algae blooms in experimental ponds but bioplastics are less harmful" has now been seen by our reviewers, whose comments appear below. In light of their advice we are delighted to say that we are happy, in principle, to publish a suitably revised version in Communications Sustainability.

We therefore invite you to revise your paper one last time to address the remaining concerns of our reviewers. At the same time we ask that you edit your manuscript to comply with our format requirements and to maximise the accessibility and therefore the impact of your work.

EDITORIAL REQUESTS:

Please review our specific editorial comments and requests regarding your manuscript in the attached "Editorial Requests Table".

*****Please take care to match our formatting and policy requirements. We will check revised manuscript and return manuscripts that do not comply. Such requests will lead to delays. *****

Please outline your response to each request in the right hand column. Please upload the completed table with your manuscript files as a Related Manuscript file.

If you have any questions or concerns about any of our requests, please do not hesitate to contact me.

SUBMISSION INFORMATION:

In order to accept your paper, we require the files listed at the end of the Editorial Requests Table; the list of required files is also available at https://www.nature.com/documents/commsj-file-checklist.pdf .

OPEN ACCESS:

Communications Sustainability is a fully open access journal. Articles are made freely accessible on publication. For further information about article processing charges, open access funding, and advice and support from Nature Portfolio, please visit https://www.nature.com/commssustain/open-access

At acceptance, you will be provided with instructions for completing the open access licence agreement on behalf of all authors. This grants us the necessary permissions to publish your paper. Additionally, you will be asked to declare that all required third party permissions have been obtained, and to provide billing information in order to pay the article-processing charge (APC).

Please use the following link to submit the above items:
Link Redacted
** This url links to your confidential home page and associated information about manuscripts you may have submitted or be reviewing for us. If you wish to forward this email to co-authors, please delete the link to your homepage first **

We hope to hear from you within two weeks; please let us know if you need more time.

Best regards,

Nandita Basu, PhD
Associate Editor, Communications Sustainability
Consulting Editor, Communications Earth & Environment
Nature Portfolio

REVIEWERS' COMMENTS:

Reviewer #1 (Remarks to the Author):

This manuscript has been carefully revised according to the reviewer's comments and meets the publication requirements of this journal. I have no other comments.

Reviewer #2 (Remarks to the Author):

The authors have sufficiently addressed my comments.

** Visit Nature Portfolio's author and reviewers' website at www.nature.com/authors for information about policies, services and author benefits**

Open Access This Peer Review File is licensed under a Creative Commons Attribution 4.0 International License, which permits use, sharing, adaptation, distribution and reproduction in any medium or format, as long as you give appropriate credit to the original author(s) and the source, provide a link to the Creative Commons license, and indicate if changes were made.
In cases where reviewers are anonymous, credit should be given to 'Anonymous Referee' and the source.
The images or other third party material in this Peer Review File are included in the article's Creative Commons license, unless indicated otherwise in a credit line to the material. If material is not included in the article's Creative Commons license and your intended use is not permitted by statutory regulation or exceeds the permitted use, you will need to obtain permission directly from the copyright holder.
To view a copy of this license, visit https://creativecommons.org/licenses/by/4.0/

Microplastic pollution induces algae blooms in experimental ponds but bioplastics are less harmful

Corresponding Author: Scott G. Morton

We thank the reviewers for their thoughtful and constructive feedback. We believe the revisions have improved the clarity, rigor, and overall quality of the manuscript. We have taken care to expand upon the discussion section and to provide essential details regarding the experimental design. We hope these changes adequately address your concerns, and we look forward to hearing your feedback.

Comments from Reviewer 1

Reviewer #1 (Remarks to the Author):

This paper examines the effects of three types of microplastics on planktonic communities. This work is interesting and has certain practical significance. The data and figures in the paper are very rich. However, it is regrettable that the mining of information from these figures is still not sufficient, especially in the discussion section. Therefore, I suggest that the paper needs further major revisions. Here are the detailed comments:

Rev. 1, Comment 1: Line 19-20: What kind of response changes? This should be elaborated in detail.

Response to Rev. 1, Comment 1: The sentence now reads, Microbial communities analyzed using 16S and 18S rRNA data exhibited 'compositional shifts in response to all three plastic types, with petroleum-derived plastic associated with the most pronounced differences across both prokaryotic and eukaryotic domains.'

Rev. 1, Comment 2: Line 25: Replace "documented" with "detected."

Response to Rev. 1, Comment 2: 'documented' replaced with 'detected'

Rev. 1, Comment 3: Line 26: Rewrote "some of earth's most remote."

Response to Rev. 1, Comment 3: The sentence now reads 'These pollutants have been detected in nearly all ecosystems, including remote and extreme environments such as deep sea trenches and arctic sea ice [4,5]'

Rev. 1, Comment 4: Line 44: "particularly in freshwater ecosystems, but see.?" This does not seem to be a complete sentence.

Response to Rev. 1, Comment 4: The sentence has been updated and now reads as 'Yet, despite the ubiquity of microplastics in the environment, few studies have examined their broader ecological impacts, particularly in freshwater ecosystems, though exceptions can be found [19,30]'

Rev. 1, Comment 5: Line 52: The first mention of TPU should include its full name. And why were TPU 181 and TPU FC2.1 chosen as materials?

Response to Rev. 1, Comment 5: The sentence now reads as '**We used one conventional petroleum-derived thermoplastic polyurethane (TPU), Elastollan, chosen for its commercial relevance; and two biodegradable TPU products, TPU 181 and TPU FC2.1, which represent emerging bio-based alternatives.' We further describe the rational just below these statements on lines 62-67.

Rev. 1, Comment 6: Line 71: (Est.=3.451)

Response to Rev. 1, Comment 6: Corrected to (Est.=3.451)

Rev. 1, Comment 7: Line 75: "Higher concentrations" refers to what level? In Figure 1, it can be observed that in the two biodegradable plastic treatment groups, when the concentration of microplastics is greater than 0.023 g/L, the concentration of chlorophyll a also shows high values.

Response to Rev. 1, Comment 7: We thank the reviewer for pointing out the need for additional clarity the section now reads 'As a proxy for primary producer biomass, chlorophyll-α concentrations varied notably across plastic treatments over time (Fig. 1). The best- fit model (Model 3) included significant effects of date, plastic type, and concentration, along with their interactions (Extended Data Table 1). Both bioplastics (TPU 181 and TPU FC2.1) yielded significantly lower chlorophyll-α (Est. = -0.728, -0.570, respectively) compared to the petroleum-derived plastic (Est. = 3.451) (Fig. 1, Extended Data Table 2). The model revealed significant non-linear effects of 'plastic concentration' and 'sampling date' on chlorophyll-α concentrations, varying distinctly across plastic types with the model explaining 66% (R^2 = 0.212) of the deviance in

chlorophyll-α concentrations. Though all plastic types exhibited variable algal bloom occurrences at concentrations ≥ 0.041 g L^{-1}, tanks dosed with Elastollan above 0.013 g L^{-1} maintained consistently elevated chlorophyll-α throughout the second half of the experiment, an effect never seen at lower Elastollan concentrations.

Rev. 1, Comment 8: Line 84-90: You repeatedly mention "lower" and "higher." However, this is a qualitative description. It should be noted that you are describing the results, so you need to provide some specific numbers instead of just describing whether they are lower or higher.

Response to Rev. 1, Comment 8: The section now reads 'Elastollan-treated tanks exhibited significant zooplankton biomass deficits relative to both TPU 181 and TPU FC2.1 across a progressively narrowing concentration range over time (Fig. 2). On individual sampling dates, biomass variation was best explained by a model which included a fixed effect of plastic type and plastic-type-specific nonlinear effects of plastic concentration, indicating interactive effects between plastic type and concentration (Extended Data Table 3; Supplementary Figure 6). On Day 0, prior to microplastic exposure, zooplankton biomass showed a statistically significant but visually weak nonlinear relationship with microplastic concentration ($p = 0.026$; Extended Data Table 4). Since plastics had not yet been added to the system, this effect likely reflects random baseline variation, supported by separate analyses showing no significant differences among treatments at Day 0 in total biomass ($p = 0.39$), total abundance ($p = 0.43$), Shannon diversity ($p = 0.647$), or community composition ($p = 0.928$). The model including a single smooth term for plastic concentration, had the lowest AIC on day 0 (Extended Data Table 3), suggesting that observed variation likely reflected random baseline differences rather than treatment effects. After the addition of plastic, the effects of plastic type and concentration were significant predictors of zooplankton biomass (Extended Data Table 4). Across sampling dates, Elastollan consistently reduced zooplankton biomass relative to both bioplastics, with the magnitude of this effect increasing at higher concentrations (Extended Data Table 5). On Day 36, model-predicted biomass in Elastollan-treated tanks was approximately 65% lower than TPU 181 and 69% lower than TPU FC2.1. These differences grew over time, reaching 71–79% lower biomass by Day 56, and remaining 71–73% lower by Day 97. On Day 36, significant reductions in ln-biomass were observed at concentrations as low as 0.008 g L^{-1}, but by Day 97, only concentrations ≥ 0.072 g L^{-1} remained significant for TPU 181 and concentrations ≥ 0.126 g L^{-1} for TPU FC2.1. This shift toward higher concentration thresholds, despite consistently large biomass deficits, suggests that Elastollan's

suppressive effect on zooplankton biomass weakened as the experiment progressed (Extended Data Table 5).'

Rev. 1, Comment 9: Line 150: Rewrite "in the upper half of the plastic concentration range."

Response to Rev. 1, Comment 9: The sentence has been updated for clarity and now reads as 'In each of the two bioplastics treatments, blooms developed in tanks treated with plastic concentrations = 0.041g/L, and additionally at 0.126g/L with TPU FC2.1, but not in any other tanks.'

Rev. 1, Comment 10: Line 227: Why is it 58:25:17?

Response to Rev. 1, Comment 10: We added two clarifying sentences after the particle size ratio is presented which read 'The particle size ratio added to each pond reflected the relative abundances of each size class available following processing and sieving of the materials. This ratio was standardized across all treatments to maintain consistency.'

Rev. 1, Comment 11: Line 228: Since the experimental water came from municipal water, were the background values of the water body in the municipal water measured? For example, concentrations of nitrogen, phosphorus, and chlorophyll a, or environmental parameters (temperature, DO, pH, conductivity, TDS, and salinity), because the nutritional status of these waters can affect plankton.

Response to Rev. 1, Comment 11: Line 233 – Added 'Background environmental parameters were measured prior to the start of the experiment (Supplementary Table 2), with additional water quality metrics reported by the City of San Diego Public Utilities Department (City of San Diego Public Utilities Department, 2023).'

Variable	*Min*	*Mean*	*SD*	*Max*
Temperature (°C)	12.2	12.443	0.104	12.6
Dissolved O_2 (%)	88.3	100.763	5.273	113.9
Dissolved O_2 (mg/L)	9.37	10.726	0.567	12.22
Conductivity (µS)	0.64	0.672	0.024	0.754
TDS (g/L)	0.416	0.437	0.015	0.49
Salinity (ppt)	0.31	0.329	0.013	0.37
pH	9.2	9.433	0.152	9.78

Chlorophyll-a (µg/L)	9.732	29.985	15.907	71.562

Added City of San Diego Public Utilities Department. Annual Drinking Water Quality Report 2023. (City of San Diego, 2024) to citation list.

Rev. 1, Comment 12: Line 231-232: Why were different amounts added at different times?

Response to Rev. 1, Comment 12: Thank you for pointing out this insufficiency in explanation. We have added 2 additional sentences to clarify. The paragraph now needs; 'Each experimental pond was filled with municipal water (Nov 7, 2022), San Diego, CA and seeded with a concentrated mixture of live zooplankton, phytoplankton and microbes. This mixture was collected using vertical tows of a >63 µm mesh plankton net from Lake Miramar (Nov 11, 2022) and Lake Murray, San Diego, CA (Nov 22, 2022, and Jan 6, 2023) with 25 mL added on Nov 11 and Nov 22, and 35 mL on Jan 6. Inoculate volume reflected variation in the total plankton yield from each field sampling event. Multiple inoculations were performed to help establish robust zooplankton populations prior to microplastic additions. To support algal growth of phytoplankton from the inoculate and ensure a sufficient food source for zooplankton, nutrients were added to each tank prior to the start of the experiment. Tanks were enriched with 25 µg/L (Dec 26, 2022) and 100 µg/L (Dec 29, 2022) of nitrogen and phosphorus (10:1 molar ratio) with sodium nitrate (NaNO□) and sodium phosphate dibasic (Na□HPO□) respectively. Plastics were then added to each mesocosm (Mar 9, 2023) at specified treatment concentrations.'

Rev. 1, Comment 13: Line 250, 257: I noticed that the sampling times for phytoplankton and microorganisms are not consistent. Why?

Response to Rev. 1, Comment 13: The sampling times for chlorophyll-α and zooplankton (bulk measurements) versus the microbial communities (16S/18S rRNA sequencing) were not perfectly aligned due to logistical constraints, including personnel availability and sample processing time requirements. While ideal alignment would have been preferred, the conclusions drawn in the manuscript do not rely on direct statistical comparisons between these datasets. Instead, each dataset contributes complementary insights into treatment effects over time. Although sampling was staggered, we believe the timing was sufficient to detect relevant trends within each biological group and does not affect the validity of our overall interpretations.

Rev. 1, Comment 14: The data and figures in the paper are very rich. However, the discussion lacks in-depth analysis. For example, your figures show rich information about microorganisms. However, it is regrettable that the discussion does not cover these results, and thus lacks some eye-catching content for readers. What you have discussed are more general findings. I suggest that the discussion section needs to be further explored in combination with your results.

Response to Rev. 1, Comment 14: We have expanded the discussion section to include a more detailed interpretation of our microbial community results (beginning on Line 276). Specifically, we now describe key bacterial and eukaryotic taxa that were differentially enriched across treatments (e.g., Ochromonadaceae, Tetramitia, Acetobacteraceae) and discuss their ecological roles. We additionally highlight how Elastollan influenced both phototrophic and heterotrophic microbial assemblages compared to either bioplastic, suggesting trophic restructuring of microbial communities. These additions emphasize the functional implications of our 16S/18S results and clarify how plastic type and concentration shaped microbial responses over time.

Rev. 1, Comment 15: The clarity of Figure 3 is very poor, and the font is also too small to obtain information. The same applies to Extended Data Figures 1, 2, 6, and 8. Additionally, consider adding 95% confidence ellipses for each treatment group. The distinction between these three colors is not significant enough, and I suggest changing the colors.

Response to Rev. 1, Comment 15: We have updated the color scheme to make colors more distinct from one another. We have also increased the font in Fig 3 and Ext Data Figures 1, 2, 6, and 8. In regard to adding ellipses, we had previously placed 95% confidence ellipses associated with each treatment on the plots, however this cluttered the figures and made interpretations less visually apparent.

Rev. 1, Comment 16: Line 863: "with numbers 0–9 indicating increasing experimental concentrations of microplastics." The nine concentrations should be specified.

Response to Rev. 1, Comment 16: We have added an additional sentence to the figure caption to include the specific concentrations which reads 'The specific concentrations (g/L) corresponding to labels 0–9 are as follows: 0 = 0.000, 1 = 0.004, 2 = 0.010, 3 = 0.013, 4 = 0.023, 5 = 0.041, 6 = 0.072, 7 = 0.126, 8 = 0.220, and 9 = 0.385.'

Comments from Reviewer 2

Reviewer #2 (Remarks to the Author):

The authors have conducted a thorough study that investigates the response of different trophic levels to marine debris plastics. Often sequencing is only on the bacterial level in this field, but a strength of this paper is that they considered algae and protists. They also compared a bioplastic to a petroleum-based plastic in the same category. Some weaknesses of the paper include some missing methodological detail especially around volume, shape and growth conditions of the ponds and some plastic properties. It is unclear if no plastic controls were conducted and/or considered in the statistical analyses, and it is also unclear if replicates were conducted for each concentration. Also, some main figures, extended data and supplementary materials do not have a call-out in the manuscript which required some guessing of what data is the evidence for certain statements. Further some things are not defined at their first mention like what the different plastics actually are or what model 3 is (all in methods which is after intro and results).

Introduction section:

Rev. 2, Comment 1: - Third paragraph, last sentence is incomplete.

Response to Rev. 2, Comment 1: The last sentence in the third introduction paragraph is a complete sentence – no changes needed. If the reviewer was referring to the last sentence of paragraph two, this was also mentioned by reviewer 1 and corrected (See Rev 1, Comment 4).

Results section:

Rev. 2, Comment 2: - Chl-a: Figure 1 call-out is missing. Further, it is very confusing to the reader when the first sentence of the results section is a stats result but does not provide context about what experiment was done, what was observed or what results that stats are analyzing.

Response to Rev. 2, Comment 2: An introductory sentence was added to provide context and Fig. 1 referenced. The section now reads 'As a proxy for primary producer biomass, chlorophyll-α concentrations varied notably across plastic treatments over time (Fig. 1). The best fit model included plastic type as a fixed effect, with plastic type-specific smooth terms for time and concentration (Extended Data Table 1). Both bioplastics (TPU 181 and TPU FC2.1) yielded significantly lower chlorophyll-α (Est. = -0.728, -0.570, respectively) compared to the petroleum-derived plastic (Est. = 3.451) (Extended Data Table 2).'

Rev. 2, Comment 3: - Biomass: Did they observe any biofilm formation that might affect biomass readings or are the ponds so big and the concentration of plastics so small that the amount of biomass on the plastic is likely to be negligible?

Response to Rev. 2, Comment 3: We did not observe biofilm formation on the plastics during the experiment. Given the large volume of the experimental ponds and the small particle sizes used, any biomass associated with plastic surfaces would be negligible compared to the total biomass in the system. We therefore did not expect biofilm on plastics to influence our biomass measurements.

Rev. 2, Comment 4: - Microbial Community: The results section often reports a statistical finding without putting it in the biological context. For instance, 'plastic concentration was significant in the 16S dataset but not 18S.' What was observed to change in the communities with plastic concentration? Does this include a no-plastic control?

Response to Rev. 2, Comment 4: We have revised the results section to include biological context regarding the observed effect of plastic concentration on microbial communities and additionally throughout the results section. Yes, all analyses include a unique no plastic control pond associated with each plastic type.

Discussion Section:

Rev. 2, Comment 5: - I would recommend included a schematic that describes the layers of interactions they think are happening. I agree with the interpretation but a visualization of the trophic levels and differential effect of plastics would be helpful.

Response to Rev. 2, Comment 5: We appreciate the suggestion to include a schematic summary of interactions. However, after consideration, we feel that adding such a figure would not substantially increase the clarity of our results, as the system contains relatively few trophic levels and the interactions are already conveyed in detail through the individual main figures. We are concerned that a schematic could risk oversimplifying or misrepresenting the observed patterns.

Rev. 2, Comment 6: - In the second paragraph they describe HABs as unpredictable but in the next sentence they say that they found things that always happen, that phrasing felt contradictory.

Response to Rev. 2, Comment 6: We thank the reviewer for this comment and have updated the paragraph to flow more logically and now it read as 'Harmful algal blooms

(HABs) are stochastic, unpredictable events typically emerging from nonlinear interactions, environmental drivers, and biological communities [44–46]. In our experiment, dense algae concentrations always developed in tanks with high concentrations of Elastollan, but never in the three tanks with the lowest levels of microplastics. However, these blooms were relatively asynchronous, following differing trajectories, and their intensity was not clearly related to a dose-response relationship above the lower threshold. In each of the two bioplastics treatments, blooms developed in tanks treated with plastic concentrations = 0.041g/L, and at 0.126g/L with TPU FC2.1, but not in any other tanks. Our results indicate that microplastics may tip the balance of conditions in favor of algal blooms but that stochasticity still plays a large role in when and where such blooms occur.' We feel these changes clearly introduce the stochastic nature of algal blooms, while also highlighting the consistent patterns observed in our study and acknowledge the remaining unpredictability in bloom dynamics observed in our results.

Rev. 2, Comment 7: - In the third paragraph a discussion of how the no plastic control behaved would help to interpret the acute exposures. Toxicity in these cases is often from the water soluble additives in commercial plastics leaching and disrupting the community. Then once those are consumed, it is more the surface interactions with plastics or ingestion if the pieces are small enough relative to the organism.

Response to Rev. 2, Comment 7: We have added this statement to the third paragraph of the discussion 'Importantly, microbial communities in no-plastic control tanks remained relatively stable across sampling periods, indicating that the observed shifts were not simply due to background temporal variation but were likely driven by plastic exposure (Supplementary Table 15).' We have included the referenced table in the supplementary information.

Supplementary Table 1 Community metrics for no-plastic control tanks

Dataset	*R^2*	*F*	*p*
Zoop Abundance			
PERMANOVA	0.270	1.666	0.095
ANOVA		2.511	0.136
Shannon		0.816	0.472
16S			
PERMANOVA	0.240	1.898	0.053
ANOVA		1.000	0.397
Shannon		0.290	0.753
18S			

PERMANOVA	0.200	1.421	0.250
ANOVA		0.411	0.673
Shannon		0.855	0.452

Results show variation over time in control tanks based on PERMANOVA (community composition), ANOVA on total abundance and Shannon diversity. R^2, F-statistics, and p-values are reported for each test. These analyses assess background temporal variation in the absence of plastic exposure.

Materials and Methods Section/throughout:

Rev. 2, Comment 8 - Controls: In some figures it looks like a no plastic control was run, in other figures it is absent. It also unclear if included in stats. It is very important to compare to a no plastic exposure condition. A table or diagram of the experimental set up and all conditions tested would be very helpful.

Response to Rev. 2, Comment 8: All figures have an explicit 'no plastic' designation or a 0-concentration level visually represented on the plots. We have also added a clarifying paragraph at the start of the statistical tests section in the methods which reads 'We randomly assigned one unique control tank (0 µg/L microplastic) to each plastic type at the outset of the experiment to maintain a balanced factorial design. This approach ensured that each level of plastic type in the statistical models included a corresponding 0-concentration reference, avoiding confounding between plastic type and concentration. While some natural variability among controls was observed (as is expected in outdoor systems), these differences were minor relative to treatment effects and were explicitly accounted for in all statistical models.'

While we agree that a visual summary of the experimental design could be helpful, we believe the clarified description of methods provides sufficient detail for readers to understand the structure of the experiment. The design is essentially 3 replicated concentration gradients and a visual representation would not hold too much detailed information. We hope this is better explained in the text but can work to provide a visual representation if still needed. As such, we have opted not to include an additional table or diagram at this time.

Rev. 2, Comment 9: - Ponds: More information about the ponds/mesocosms (called both in manuscript) must be given. What is their volume and shape? What are they made out of? Are they outside? How is light controlled? Are they stirred, at what rate? Temperature etc? Freshwater, brackish or salt water? Were the pond conditions arranged in a randomized design?

Response to Rev. 2, Comment 9: The naming has been changed to always say experimental pond, the mesocosm designation has been removed to reduce confusion. We have included greater detail on pond materials and characteristics, set-up, and location within the first paragraph of the methods section.

Rev. 2, Comment 10: - Replicates: Were replicates conducted or how do they know the observed phenotypes that have many stats run on them are reproducible or reliable?

Response to Rev. 2, Comment 10: We acknowledge that the experiment used a continuous concentration gradient without true biological replication at each concentration level. The experiment was intentionally structured as an unreplicated gradient to detect continuous, dose-dependent responses to plastic concentration. Each unique concentration level serves as a distinct treatment in the gradient, and our models (e.g., GAMs) are specifically suited to identify non-linear patterns along such gradients. To ensure statistical robustness, we used models that account for heteroscedasticity and potential autocorrelation across the gradient. Furthermore, confidence intervals and effect sizes were evaluated alongside p-values to avoid overinterpretation of individual statistical tests.

Rev. 2, Comment 11: - Polymer properties of 3 plastics chosen and justification for why these 3 of the thousands of plastics: Please provide a justification for why these 3 plastics out of the thousands of polymers that exist and pollute waterways. Why did the choose two different bioplastics and what are different about these? Do all 3 plastics have similar size, shape, thickness, crystallinity, surface roughness, density etc? We and others globally have found that interactions are very polymer and microbe specific. In order to compare two plastics of bioplastic vs conventional, they should be as similar as possible apart from the origin. The authors also speak about how these plastics will or won’t break down but the plastics are not recovered or tested in this paper limiting their ability to comment on if that was a factor in the observed phenotypes.

Response to Rev. 2, Comment 11: We have included additional details and references regarding the makeup of each plastic type. Regarding the plastics ability to breakdown – We used information from the Elastollan datasheet describing its limited degradation and previous studies showing related bio-based plastics degradation in compost. Though we explicitly acknowledge that we did not directly measure degradation of plastics in the manuscript. All of these additional details can be found in the first paragraph of the methods section.

Rev. 2, Comment 12: - Location of the plastics in the tank and sampling depth and times: These plastics are more dense than water- Do they sink in these tanks? Is there stirring? Are the plastics in the same part of the water column with the algae? How deep into the tanks is the sampling occurring? Was sampling done at the same time of day to control for diurnal variability in community composition?

Response to Rev. 2, Comment 12: Line 330: added 'which captured the entire water column', Line 338: added 'in synergy with zooplankton samples, water was taken'

Rev. 2, Comment 13: - Zooplankton identification: no reference is given for 'available published keys'

Response to Rev. 2, Comment 13: We have now included the citation to the zooplankton key in the methods section.

Statistical analyses:

Rev. 2, Comment 14: - If there are no replicates, some explanation should be given for using the concentration gradient as pseudo-replicates or how the stats and models they chose are still appropriate to use without biological replicates. If at least a different no-plastic pond was run for each plastic, did each of the no-plastic ponds behave similarly?

Response to Rev. 2, Comment 14: We included a 0.00 g/L plastic treatment as the control condition across all plastic types, with one unique 0-concentration tank assigned per plastic type. Each concentration level, including 0 g/L, was treated as a unique treatment in a continuous exposure gradient design, and not as a biological replicate. Our statistical models (e.g., generalized additive models) were chosen specifically to handle this structure, incorporating plastic concentration as a continuous predictor and allows for nonlinear responses via smooth terms. This approach avoids treating the gradient as replicated treatments. Additional, plastic type was included as a fixed effect, and the associated 0-concentration control served as a reference level within each type. This allowed us to estimate concentration-dependent deviations relative to type-specific baselines. Natural variation among these outdoor ponds was minor compared to treatment effects and was explicitly accounted for in all models. We have clarified this design rationale and control structure in the revised methods section.

Rev. 2, Comment 15: - For all of the Data Tables reporting stats, please define the column headings in footnotes at least once. Also they say model selection but I don't

see the different models represented in the charts. Are the ones displayed model 3 only?

Response to Rev. 2, Comment 15: We have now added footnotes to the tables defining all column headings. Regarding model selection, the AIC reporting set of tables presents all candidate models and their AIC values, with the best-fit model having delta AIC indicated with bold font. The main figures and tables reporting findings present results from the best-fit model only. We have clarified this in the figure and table captions to ensure the displayed results correspond to the best-fit model identified through AIC comparison.

Rev. 2, Comment 16: - It wasn't clear if extended data figures 1 & 2 were called out in the text or what they add beyond what is described in Figure 3.

Response to Rev. 2, Comment 16: Both Extended Data Figures 1 and 2 are cited in the text (Line 179). These figures present overlaid biplots for family-level taxa that are not shown in Figure 3 and add taxonomic identities for the ten most abundant taxa, providing finer-resolution context to the patterns displayed in the main figure.

Rev. 2, Comment 17: - For extended data figure 3: Are these normalized to no-plastic?

Response to Rev. 2, Comment 17: The data in Extended Data Figure 3 were not previously normalized to the no-plastic controls. We have now applied this normalization and updated the figure, methods and discussion accordingly.

Rev. 2, Comment 18: - Synthesis of TPU 181: methods are missing source of algae-based polyester-polyoyl. Also it states the mechanical properties were tested but not reported nor compared to the other plastics.

Response to Rev. 2, Comment 18: We have updated the TPU 181 synthesis section in the supplementary text. This section now reads

'**Algae-Based Polyester-Polyol Synthesis**

The polyester-polyol used for TPU 181 was synthesized following Rajput *et al.* with modifications (Rajput et al. 2022). Briefly, a three-neck round-bottom flask equipped with a Dean–Stark apparatus, reflux condenser, and oil bath was purged and maintained under nitrogen. Commercial azelaic acid (356.4 g, Croda Inc.) and algae-sourced azelaic acid (40.3 g) were combined with 1,3-propanediol (179.2 g) as described in Phung et al. 2020. The mixture was heated to 150–160 °C and gradually raised to 180 °C over one hour. Within the first 4–5 h, water by-product was continuously removed. When approximately 80% of the water was collected, dibutyltin dilaurate (DBTDL, 0.04 wt %)

was added. The reaction proceeded for an additional 2–3 days (until desired acid and hydroxyl numbers were reached), with periodic sampling for titration.

TPU 181 Preparation

The algae-based polyester-polyol (230 g) and 1,3-propanediol (8.74 g) were dried under vacuum at room temperature for 24 h before polymerization. These were then combined with DBTDL (0.03 wt %) and preheated (75 °C) hexamethylene diisocyanate (6HDI, 42.55 g) in a speed mixer (FlackTek) at 2000 rpm for approximately 1 min. The reaction mixture was poured into molds and cured at 75 °C for 2 days to form TPU sheets.

Mechanical Property Testing

Shore A hardness testing was conducted at room temperature. The resulting TPU 181 displayed a Shore A hardness of approximately 97, consistent with typical thermoplastic polyurethanes.'

We have additionally added a sentence in the introduction describing Shore A hardness for all three plastic types.

Rev. 2, Comment 19: - Please make sure all supplementary figures are called-out in the text.

Response to Rev. 2, Comment 19: All supplementary material is now called out in text.

Notes on additional changes to manuscript:

Line 459: The verbal description of model 4 was incorrectly phrased and has now been updated.

Supplementary Fig 7 now has blank bars for 3 ponds on day 0, these were removed due to low read depth. This did not change the results as the tanks had always been removed from the analysis, however the original visualization had not removed them. This is also described in the methods section Line 425 now reads 'Three samples from day 0 were removed from the analysis due to their extremely low read depths.'

Abundance and biomass tables and figures have been fully updated following a correction in the code, which had previously duplicated control tanks and assigned them to each plastic type. This error has been fixed to reflect the original experimental design, where a single unique control tank was assigned to each plastic type, consistent with the rest of the dataset.

Chlα, NPP, and ER tables have been updated to reflect a change in data transformation of the plastic concentration variable. All models now use a log1p transformation (i.e., log(x + 1)) to account for zero values. This ensured consistency across metrics.
